# Global Patterns of Evolutionary Distinct and Globally Endangered Amphibians and Mammals

**DOI:** 10.1371/journal.pone.0063582

**Published:** 2013-05-15

**Authors:** Kamran Safi, Katrina Armour-Marshall, Jonathan E. M. Baillie, Nick J. B. Isaac

**Affiliations:** 1 Department for Migration and Immuno-ecology, Max Planck Institute for Ornithology, Seewiesen, Germany; 2 Department of Biology, University of Konstanz, Konstanz, Germany; 3 Institute of Zoology, Zoological Society of London, London, United Kingdom; 4 Imperial College London, Berkshire, United Kingdom; 5 Natural Environment Research Council, Centre for Ecology and Hydrology, Oxfordshire, United Kingdom; University of Kent, United Kingdom

## Abstract

**Background:**

Conservation of phylogenetic diversity allows maximising evolutionary information preserved within fauna and flora. The “EDGE of Existence” programme is the first institutional conservation initiative that prioritises species based on phylogenetic information. Species are ranked in two ways: one according to their evolutionary distinctiveness (ED) and second, by including IUCN extinction status, their evolutionary distinctiveness and global endangerment (EDGE). Here, we describe the global patterns in the spatial distribution of priority ED and EDGE species, in order to identify conservation areas for mammalian and amphibian communities. In addition, we investigate whether environmental conditions can predict the observed spatial pattern in ED and EDGE globally.

**Methods and Principal Findings:**

Priority zones with high concentrations of ED and EDGE scores were defined using two different methods. The overlap between mammal and amphibian zones was very small, reflecting the different phylo-biogeographic histories. Mammal ED zones were predominantly found on the African continent and the neotropical forests, whereas in amphibians, ED zones were concentrated in North America. Mammal EDGE zones were mainly in South-East Asia, southern Africa and Madagascar; for amphibians they were in central and south America. The spatial pattern of ED and EDGE was poorly described by a suite of environmental variables.

**Conclusions:**

Mapping the spatial distribution of ED and EDGE provides an important step towards identifying priority areas for the conservation of mammalian and amphibian phylogenetic diversity in the EDGE of existence programme.

## Introduction

The current ‘biodiversity crisis’ driven by anthropogenic action has led to a rate of species loss of up to a thousand times greater than that of background extinction [1]–[3]. One of the most significant issues now facing conservationists is how to best allocate limited resources for the best conservation outcome [4], given the uneven global distribution of biodiversity [5], [6]. The main question considered when defining global conservation priorities is ‘which geographical regions should be protected so as to maintain maximum biological diversity?’ [7]. Major institutional strategies of global conservation prioritisation focus on counts of irreplaceable and/or vulnerable species, and cover large portions of the earth’s land surface [5]. The priority regions identified by these strategies represent frameworks within which to allocate funding to national and local conservation projects.

Species richness approaches are limited by their failure to take into account the ecological role of species in communities and the different contributions they make to ecological communities. Biodiversity value may thus be better estimated by its contribution to evolutionary history, where more evolutionarily distinct species have higher value [8], [9]. Preservation of phylogenetic diversity allows scientists and conservationists to maximise information preserved within fauna and flora [9], [10] (but see also [11]). The fact that evolutionarily distinct species generally have more divergent traits [12] suggests they might play a disproportionate role in ecosystem functioning [8], [13]. Atkinson [14] observed that “given two threatened taxa, one a species not closely related to other living species and the other a widespread and common species, it seems reasonable to give priority to the taxonomically distinct form”.

An additional argument for considering phylogenetic information in conservation is that extinction risk is not phylogenetically random [3]. Closely related species show similar threat levels; extinction risk is generally higher in species which are large, long-lived, slowly reproducing and with specialised habitats and high levels of endemism [1], [3], [9]. Species predicted to survive into the future are likely to be widespread generalists (sometimes called ‘weedy species’), replacing those considered specialised, charismatic and distinctive [9].

To date, the protection of phylogenetic diversity has not yet been incorporated into priority setting approaches employed by conservation funding agencies or NGOs. Available research has indicated that priority regions such as biodiversity hotspots contain more phylogenetic diversity than expected by species numbers alone [15]. Recent studies also suggest that the loss of phylogenetic diversity is not spatially random and that regions such as the Amazonian basin and South East Asia are losing phylogenetic diversity faster than expected by random extinction [16]. Moreover, species contributing highly to phylogenetic diversity are no more likely to receive conservation attention than average [17]. Although there are many suggested measures of phylogenetic diversity at the community level [13], [18]–[20], only a few species-based measures exist and only one has been promoted as an institutional conservation programme [1], [10]. The ‘EDGE of Existence’ programme [21] raises conservation awareness and funding for species that are both evolutionary distinct (ED) and globally endangered (GE, i.e. they are highly threatened). The algorithm for generating ED and EDGE scores has been extensively tested [1], [10], [22] but the distribution of these metrics in space has not been mapped to date.

Here, we identify priority areas based on risk of extinction and evolutionary uniqueness of species by investigating the global distribution of ED and EDGE species. Our goal is to identify the regions of the world where ED species (ED ‘zones’) and EDGE species (EDGE ‘zones’) are concentrated. We also seek to understand possible environmental factors that are correlated with high ED and EDGE, in order to shed light on the processes driving global patterns of phylogenetic diversity and threat.

## Methods

We used published ED and EDGE scores for mammals [1], [10] and amphibians [22], which we obtained from the EDGE of existence programme [21] (see supplemental online material file Data S1). The range distribution data (range maps) for all species were obtained from IUCN [23] (accessed in July 2010 http://www.iucnredlist.org/technical-documents/spatial-data) (Table 1). Each species’ range was projected into a global equal area Mollweide projection and rasterised using different raster resolution from 25×25****km to 200×200****km in steps of 25 km [24]. Previous studies showed that, when using species distribution ranges, increasing resolution (decreasing grid size) distorts the spatial patterns actually increasing the error in the correct placing of hotspots compared to coarser resolution [25]–[29]. This is because fine-scale representation of species’ distributions contain a higher proportion of false positives (‘commission errors’) than coarse ones [24]. Thus, the appropriate spatial resolution for conducting analyses of multispecies distribution patterns depends on the quality of the data used to generate those distributions [26], [27], [30]. For less known taxa such as insects and amphibians the appropriate scale is 2° (or even coarser), which roughly corresponds to 200×200 km and for mammals should not be below 1° corresponding to 100×100 km [26]. We use these scales for presenting our results, but additionally ran all analyses at each spatial resolution from 25×25****km to 200×200****km in steps of 25 km. The results across spatial scales were in general qualitatively unaffected by the resolution; the results based on the additional resolutions can be found as supplemental online material and are not presented in the main text.

**Table 1:** The number of species used in the conservation area analysis.

	Mammals	Amphibians
Species with ED scores	4754	4976
Species with EDGE scores	4416	3618

To identify the regions of the world as candidate “ED” and “EDGE zones” we employed a combination of two different strategies: a species richness based approach and a randomisation based approach. In the species richness approach we mapped species richness of the top ranking species based solely on the distributions of the highest priority species, defined somewhat arbitrarily as the top 5% of ED or EDGE scores. For each of the four data sets (mammal ED and EDGE as well as amphibian ED and EDGE) we identified the areas containing the priority species (the top 5% ranking) occurring in each grid cell.

The species richness approach, however, is a species centred concept. It prioritises areas according to the number of co-occurring high priority species, and thus allows the identification of potential areas of concentrated effort. However, it neglects a large proportion of the species and the spatial processes involved in the accumulation and maintenance of evolutionary history. Applying a randomisation approach, however, we identify regions with higher accumulated ED and EDGE scores than expected by chance. The sum of the ED and EDGE scores for any grid cell is naturally strongly correlated with species richness, so it was necessary to apply a randomisation approach that takes into account species richness. The biased contribution of species ED and EDGE scores according to the size of their ranges had to be accounted for by using a weighted random sampling. Neglecting the higher probabilities that widespread species are likely to be part of any local community would underestimate their contribution relative to species with small ranges. In combination with the fact that species with small ranges are more likely to be higher ranked in EDGE due to their higher extinction vulnerability, an un-weighted sampling would bias the estimates of the cumulative ED and EDGE scores. Therefore, for all observed values of species richness (*i* from 1 to n), we sampled 1000 times *i* species, with replacement, from the global pool of species, using a weighted sampling scheme with the probability for each species being selected proportional to the size of its geographic range. From these 1000 samples for each grid cell we derived an empirical distribution function to investigate the dispersion of the realised ED and EDGE scores. Using the empirical distribution function we derived the position (quantile) of the observed realised cumulative score (qED, qEDGE). We specifically highlight the >97.5% percentile (corresponding to a two tailed probability of p<0.05) where qED and qEDGE scores of grid cells were significantly overdispersed by being among the highest 2.5% of the randomly selected communities.

We defined ED and EDGE zones for mammals and amphibians from the intersection of the areas containing the 5% top ranking species and those areas characterised by a significantly overdispersed ED and EDGE scores respectively. Finally, we quantified the percentage of the ED and EDGE areas intersecting protected areas (data accession Feb 2013) of any level according to the World Database on Protected Areas (http://protectedplanet.net/) to assess the degree of overlap between ED and EDGE zones with existing conservation areas.

### Environmental Correlates of qED and qEDGE

If ED or EDGE scores accumulate under specific environmental conditions, predictive models could inform about mechanistic relationships between environmental conditions and the species community stability and how evolutionary history is accumulated and/or extinction risk is related to the environment. Such knowledge would allow us to make suggestions about future changes and the mitigation of extinction risk [31]. We therefore modelled the dispersion of the qED and qEDGE using a digital elevation model (WorldClim), a series of climatic variables (WorldClim), the human impact (http://sedac.ciesin.columbia.edu/wildareas/) and land cover information (Globcover 2009 V2.3). The total of 67 climatic variables representing monthly precipitation (12 raster layers), monthly minimum (12 raster layers), mean (12 raster layers) and maximum temperature (12 raster layers), as well as 19 monthly bioclimatic variables provided by BioClim (http://www.worldclim.org/bioclim) representing biologically meaningful derivatives of temperature and rainfall were downloaded at a resolution of 30 arc seconds (∼1****km). Human footprint raster and land cover were provided at a resolution of 1 km. All the rasters were aggregated and reprojected in Mollweide equal area projection to the same resolutions as the rasterised species’ range maps (from 25 to 200 km grid cell size in steps of 25 km) using R and the library “raster” [32]. The continuous measures such as human foot print or climatic variables were for reprojections bilinearly interpolated and then aggregated by taking the mean. The categorical data of land use was aggregated by assigning the modal value of the grid cells (most common land cover type) and then reprojected using the nearest neighbour assignment.

In order to reduce the high levels of co-linearity between the numerical variables (temperature, precipitation and human foot print), prior to the statistical tests, we performed a principal component analysis (PCA) reducing the data into fewer orthogonal (uncorrelated) components. The PCA was performed for each resolution separately. For all subsequent analysis we then used the first eight PCA components, representing more than 95% of the original variance. The first two PCA components contained mainly the variables associated with temperature (PCA1) and precipitation (PCA2) (see also [31]), and the remaining 6 PCAs represented complex associations of the remaining variables.

The procedure for testing for a correlation between environmental information and qED/qEDGE involved several steps of variable selection. We used generalised additive models (GAM) to predict qED and qEDGE scores as a function of the environmental layers based on 1000 randomly selected grid cells. To account for the spatial autocorrelation structure in the data, we included a smooth function using longitude and latitude of the selected grid cells in the models in the GAM; an approach that has shown to successfully account for spatial autocorrelation similar to a trend surface analysis [33]. For each raster resolution we associated qED and qEDGE from 1000 randomly selected grid cells with the PCA values based on the corresponding environmental maps with the same projection and resolution. To overcome potential biases due to basing our statistical models on a subset of 1000 grid cells (a computational limitation), we repeated each test 100 times for each grid resolution and stored the estimated slopes and intercepts of the each model. From these 100 estimates of slopes and model intercepts, we determined whether the estimated slopes significantly deviated from zero by fitting an empirical distribution function to the 100 estimates.

Finally, we retained all PCAs that, across all resolutions, had a slope significantly deviating from 0 (0.025<p>0.975), added land use as a categorical variable and reran the tests in the same way as described above.

## Results

**Figure 1:**
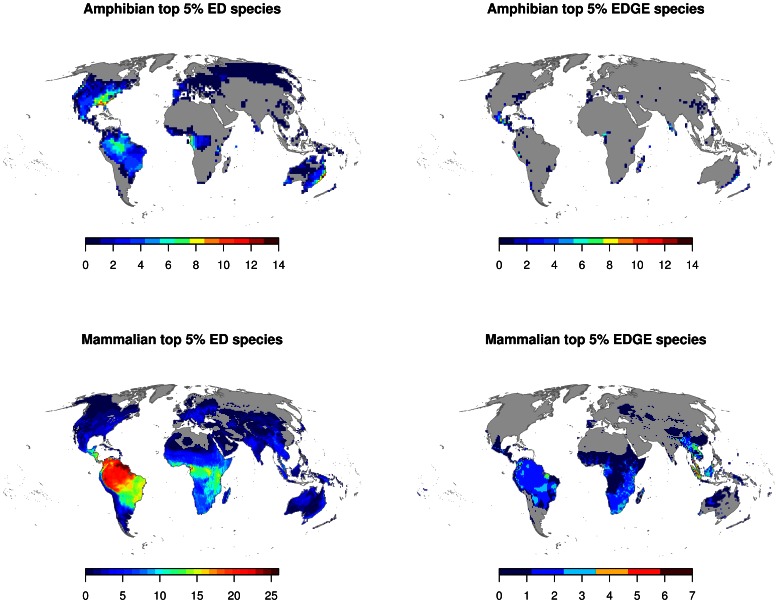
Maps of species richness of the top 5% ranking ED and EDGE species for amphibians (in a resolution of 200×200 km grid cell size) and mammals (100×100 km grid cell size).

Species richness maps of the top ranking 5% of the mammal and amphibian species indicated that these species are rarely found in large numbers in the same area (Figure 1 & Table 2). Maximum species richness values of the top 5% ED species was as low as 26 mammal species and 14 amphibian species per grid cell and broke down to 8 mammal and 14 amphibian top 5% EDGE species in one grid cell (Table 2).

**Table 2:** Land surface coverage and maximum species richness in relation to different top ranking sub sets of the mammalian and amphibian ED and EDGE assessments at all resolutions considered in the study.

		Amphibia							
	Scale (cell size in km)	25×25	50×50	75×75	100×100	125×125	150×150	175×175	200×200
	maximum species richness	12	12	13	14	12	12	12	14
**ED**	Total Area (in million km^2^)	56.22	56.62	57.26	58.03	59.11	60.68	61.71	64.00
	Proportion of land surface	0.50	0.49	0.49	0.48	0.48	0.48	0.48	0.48
	maximum species richness	5	6	5	8	9	11	13	14
**EDGE**	Total Area (in million km^2^)	2.89	3.32	3.89	4.75	5.69	6.77	8.05	9.28
	Proportion of land surface	0.03	0.03	0.03	0.04	0.05	0.05	0.06	0.07
		**Mammalia**							
	Scale (cell size in km)	25×25	50×50	75×75	100×100	125×125	150×150	175×175	200×200
	maximum species richness	26	26	26	26	26	26	25	26
**ED**	Total Area (in million km^2^ )	102.28	102.72	103.50	104.63	105.78	107.62	109.24	111.88
	Proportion of land surface	0.73	0.71	0.70	0.68	0.67	0.66	0.65	0.64
	maximum species richness	7	7	7	7	7	7	8	8
**EDGE**	Total Area (in million km^2^)	48.80	49.31	50.16	51.37	53.08	54.81	56.81	58.88
	Proportion of land surface	0.35	0.34	0.34	0.34	0.33	0.34	0.34	0.34

The entire area containing the top 5% ED species covered around 70% of the terrestrial land-surface in mammals and 50% in amphibians (Table 2). The spatial extent was smaller when extinction risk was factored in, using the EDGE scores, yet still the top 5% of the EDGE species covered an area of two thirds of the land surface in mammals and 7% in amphibians (Table 2). The grid cell size, or for that matter different thresholds than 5%, had little effect on the sizes of the areas of the top ranking species since the size of these areas were predominantly determined by the range size of the species being highest ranked (see also supplemental online material files Maps S1 & Maps S2).

### Mapping Regions of High Cumulative ED or EDGE

**Figure 2:**
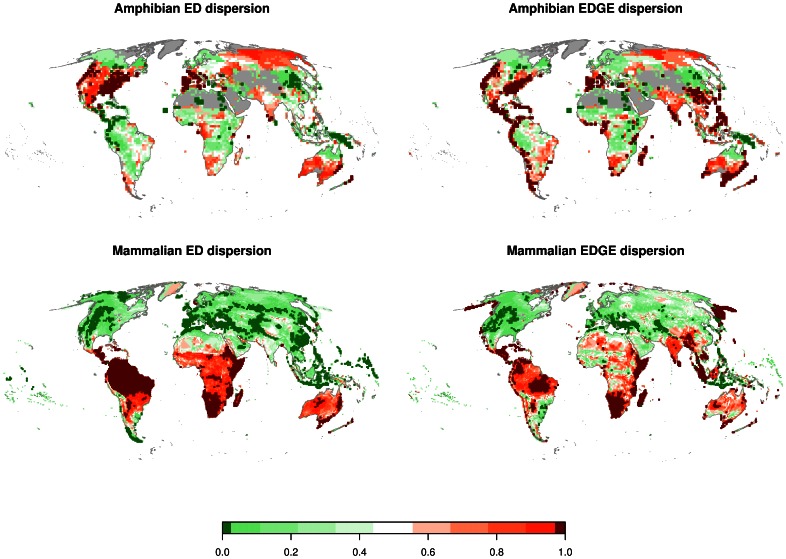
Quantile assignment of communities according to an empirical distribution function generated by 1000 randomisations. The quantile assignment indicates for each grid cell the probability of occurrence for the realised cumulative ED/EDGE value compared to 1000 randomly composed communities of equal size. Range size of the species was included in the random selection procedure to account for the fact that wide spread species are more likely to constitute a part of communities than rare and local species, and therefore a correlation between range size and ED/EDGE without taking range size into account would bias the probability distribution. The dark red and dark green areas represent areas of higher than 97.5% and lower than 2.5% probability respectively (corresponding to a p-value in two tailed statistic testing of ≤5%).

The mapping of the cumulative ED/EDGE scores revealed a somewhat contrasting pattern between mammals and amphibians (Figure 2). While the differences between ED and EDGE zones on a global scale were not large in amphibians (Figure 2), the inclusion of extinction risk (by going from ED to EDGE) altered the regional focus in mammals considerably (Figure 2). Africa and South America show high qED scores. The analysis consequently highlighted these areas as containing significantly higher cumulative ED scores than expected (qED larger than 97.5% of the random distribution; Figure 2). With the inclusion of extinction risk, the focus for areas containing the highest cumulative EDGE (irrespective of species richness) shifted towards South-East Asia. While in eastern Africa and southern Africa high levels of EDGE represent highly evolutionary distinct species communities with moderate to high levels of extinction risk, the mammal communities on the Indian subcontinent and southeast Asia are less evolutionary distinct on average but with higher risk of extinction (Figure 2).

In amphibians, the differences between qED and qEDGE are less pronounced, probably because of the higher proportion of amphibians that are globally threatened compared to mammals. In amphibians, large areas of temperate North America and Europe are consistently highlighted as important ED and EDGE areas. However, some notable differences appear in Central and South America along the Panamanian isthmus and the Andean ridge that suggest that in these areas despite moderate to low amounts of evolutionary history, species in these regions face, on average, exceptionally high extinction risk (Figure 2). The pattern of dispersion of qED and qEDGE both in mammals and amphibians remained unaffected by the resolution of the grid cells used in the analysis (see supplemental online material file Maps S3 & Maps S4).

### ED and EDGE Zones

The combination of the two approaches (qED and qEDGE >97.5% and top 5% of ED/EDGE species) was used to define the ED and EDGE zones to identify important conservation areas (spatial polygons at all resolutions intersecting the overdispersed qED and qEDGE areas with the top 5% areas can be found in supplemental material file Data S2). Mammal EDGE zones overlapped with 8.3% of the total area of the amphibian EDGE zones (total area of EDGE zones amphibians: 9.72 million km^2^ and for mammals 10.5 million km^2^ with an overlap area of 810’000 km^2^). The overlap between ED zones was even lower with only 2.3% of the amphibian zones shared with mammals (total area of ED zones amphibians: 6.04 million km^2^ and for mammals 17.29 million km^2^ with an overlap area of 140’000 km^2^). The areas of ED and EDGE zones and their relative overlap can be found for all resolutions in the supplemental material (Table S1). Also as a supplemental online resource the spatial polygons (SpatialPolygonDataFrames as defined by the library sp) for all resolutions, taxa and both prioritisation schemes ED and EDGE can be found online (Data S2).

Finally, the analysis of overlap between ED and EDGE zones revealed that 13.76±2.1% (mean±standard deviation) of the amphibian ED zones and 15.56±1.8% of the amphibians EDGE zones, across all spatial scales, were intersecting with protected areas. In mammals 29.78±1.2% of the ED zones but only 4.7±0.8% of the EDGE areas were intersecting with protected areas.

### Environmental Determinants of qED and qEDGE

The search for environmental correlates for qED and qEDGE revealed that the accumulation of ED and EDGE, and thus the quantile assignment of the realized cumulative ED and EDGE scores cannot be explained by any of the local environmental variables used in our analyses (see supplemental material for all analysis at all scales Tables S2 for the PCA based slopes and Tables S3 for the land cover analysis). Although some of the variables included in the models turned out to be significant predictors (p<0.05) at some spatial scales, none of the environmental variables was consistently influencing the distribution of qED or qEDGE globally. Even in those instances where a combination of specific spatial scale and environmental variables suggested a significant slope, the effect size was usually very low and close to zero.

## Discussion

Prioritising EDGE species for conservation draws attention away from the aesthetic and charismatic value that appears to drive many existing conservation efforts [34], and points it toward protecting evolutionary heritage. Combining geographic and phylogenetic information can identify “cradles” and “museums” of diversity; where diversity is generated and where it persists [9]. Such information would contribute to the establishment of a spatial approach to the preservation of evolutionary history. Rapidly speciating groups are occasionally prioritised above phylogenetically distinct taxa, based on the premise that these groups will speciate rapidly following an extinction episode, so replacing lost biodiversity. However, lineages showing rapid diversification rates have close relatives and are unlikely to be regarded as priority taxa based on our current choices for rarity, endemism and distinctiveness, as they represent a small proportion of unique evolutionary history [3], [8], [9].

### Historical Biogeography

The global distribution of top-ranking ED and EDGE species is crucial for addressing questions about why and how communities are composed of top ranking species and are found where they are. The highest accumulation of top mammal species ranked in terms of their EDGE score was found mainly in various African countries, South-East Asia and the Indian subcontinent, Australia and South America (see also [16]). Conservation resources would therefore be best allocated among the countries in these regions to protect mammal species with the highest EDGE scores. Countries associated with top-ranking ED species richness were found to be considerably different to those prioritised by high EDGE scores for mammals. These differences between the global distribution of high-scoring ED and EDGE mammals are the consequence of the addition of extinction risk into the prioritisation scheme [16], [35]–[37]. The distribution of ED zones are a representation of the historical biogeography in terms of continental fragmentation, vicariance and colonisation, as well as the isolation of continents and regions [38], [39]. High levels of ED are thus presumably the result of the presence of earliest marsupials and placental mammals in the Americas originating either in Africa or Gondwana [38].

Species found in Madagascar also have relatively high ED scores since Madagascar has long been an isolated island, separating from India around 90 million years ago [38], [40]. It is therefore reasonable that species found in Madagascar are top-scoring ED and EDGE species since the island contains high proportions of endemic and restricted range species [41], [42], a trait characteristic of threatened species. High scoring EDGE species particularly in Madagascar are greatly threatened by high levels of deforestation, human persecution, urbanisation and agricultural intensification [42], and therefore one would expect the combination of threat and long-term isolation to result in high EDGE.

Amphibians demonstrate high philopatry and low individual mobility [43], [44], therefore one would expect regions of high amphibian ED to coincide even more than mammals with their historical biogeography [44]. The biogeographic and historical origins of amphibians (and indeed mammals) are the subject of some debate, yet for the purpose of this paper, results are discussed in conjunction with the theories of Feller and Hedges [45]. Extant amphibians belong to one of three orders: Anura (frogs), Caudata (salamanders) and Gymnophiona (caecilians). Both salamanders and caecilians appeared during the Jurassic period, coinciding with the break-up of Pangaea around 195-157 million years ago [44], [46]. Salamanders are believed to have originated in Laurasia, and caecilians in Gondwana (but later reported in Laurasia also). Frogs are thought to have existed during the Paleozoic, when the supercontinent Pangaea was still complete, splitting into two suborders possibly due to the formation of Laurasia and Gondwana, with one suborder found on each supercontinent [45]. This theory could explain the high levels of amphibian evolutionary distinctiveness exhibited in North America and Cameroon. Should the conservation objective be to protect the oldest and most phylogenetically diverse amphibian species, efforts should be concentrated mainly in the United States. There are possible strong effects of historical and physical features (i.e. glaciations and mountain ranges) that may have restricted early amphibian dispersal, particularly in North America [45]. High amphibian ED appears to skirt around the Appalachian Mountains in the United States, indicating a possible dispersal barrier that may have led to high evolutionary distinctiveness in this region.

EDGE zones for amphibians occur mostly in Central America (Costa Rica, Mexico and Guatemala) as well as Australia, China and Cameroon. The shift of importance in ED from North to Central America, China and Australia with the addition of threat (EDGE) is likely due to species’ vulnerability in these areas to high levels of enigmatic decline, over-exploitation and reduced habitat [47]. When considered apart from the Australasian-Oceanic realm, both Australia and New Zealand exhibit a high proportion of unexpected decline [47]. Overexploitation and habitat reduction is highest in East and South-East Asia (also high in West Africa, and the Caribbean). Central America shows concentrated enigmatic decline, particularly in Mexico and Costa Rica [47]. These findings show high concordance with the distribution of highest-ranking EDGE amphibian species that are under significant threat from the above. It is important to note the dramatic spread of fungal disease chytridiomycosis as a prominent cause of decline [47]–[49].

### Patterns of Species Richness, Threat and Rarity

**Figure 3:**
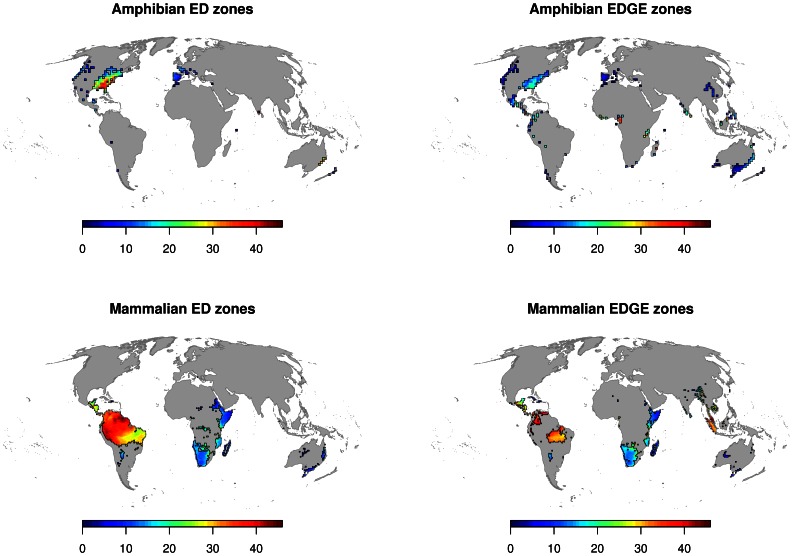
Areas for which the randomisation procedure indicated a significantly higher realised cumulative ED/EDGE scores (≥97.5 percentile) and the top 5% of the ED/EDGE species co-occur.

Species richness is, by the mathematical nature of addition, positively correlated with cumulative scores of phylogenetic and other alternative diversity measures [12], [19]. Therefore, without the randomisation approach applied here, high cumulative score areas would simply be a reflection of high species richness areas. Investigating overdispersion in qED and qEDGE, however, highlights areas where limited conservation resources would be best spent to protect the highest concentration of phylogenetic diversity irrespective of species richness. However, concentrating conservation efforts for ED and EDGE species solely on areas with overdispersed cumulative scores might not be wholly beneficial and species richness should be considered after all to prioritise among the ED and EDGE zones. Invested conservation effort could have a wider beneficial impact in species rich communities; the establishment of protected areas in species rich areas could allow the protection of ecosystems with higher complexity and potentially with a higher ecosystem value. We defined ED and EDGE zones therefore as areas containing a significantly large amount of accumulated ED or EDGE scores, which at the same time represent the highest ranking species by adding the area inhabited by the top 5% ranking species. As such, they do not represent the areas required to create complementary reserve networks (e.g. [31]), but rather identify regions of the world where priority species are concentrated, much like the original definition of the biodiversity hotspot [51]. In addition to species richness as shown in figure 3, we suggest to further prioritize the planning of conservation areas for ED and EDGE zones such that the overall effect for conservation is maximized by taking into account complementarity-based approaches, something that we did not consider here [52], [53].

### ED and EDGE Zones

Global richness patterns are often described as similar for birds, mammals and amphibians [54], promoting their use as indicator groups for general species hotspots [55]. This is not the case for ED and EDGE mammals and amphibians, whose zone distributions have considerably low overlap. Our findings indicate that the relevant ED and EDGE zones for amphibians and mammals do not always overlap, indicating the potential shortcomings of a “silver bullet” strategy [51] at least in some areas in the world for the combined protection of mammals and amphibians (Figure 2 & 3). This reinforces the conclusions of Grenyer et al. [36], who found low congruence between rare and threatened species of birds, mammals and amphibians but high cross-taxon congruence for total species richness [35], [36]. Low cross-taxon congruence between ED and EDGE zones in mammals and amphibians is probably a result of different biogeographic histories and the pronounced differences in the range sizes between mammals and amphibians. In addition, mammals and amphibians tend to be threatened by different drivers [41], [47], [56].

The concept of biodiversity hotspots [7], [51] receive a large proportion of global conservation funds [4], [5]. In cases of overlap between established biodiversity hotspots and ED and EDGE zones, high-ranking ED and EDGE species occurring within these zones can only be benefiting from existing conservation management schemes indirectly. This benefit, however, may be counteracted by species currently neglected in the ED or EDGE zones that are considerably isolated from current conservation approaches or are not included in the species targeted efforts within hotspots. Further analysis is necessary to assess congruence of ED and EDGE zones with other global and regional areas relevant for conservation such as high-biodiversity wilderness areas [57], or Frontier Forests [58]. As previously mentioned, it should be considered that even if large overlap between ED and EDGE zones and alternative global prioritisation areas are found, the benefit to each individual ED/EDGE species might still be questionable due to the large number of species generally encompassed within ‘hotspots’. Yet unlike in the purely area based approaches, the fact that the contribution of each species to the locally accumulated ED or EDGE can be quantified, allows protection and conservation measures to have a species targeted dimension, in addition to the purely spatial prioritisation that can perhaps increase the potential for successful conservation action.

Although ED and EDGE zones found in places like Madagascar (for mammals) and Central America (for amphibians) have high importance in terms of phylogenetic diversity, their inclusion in other conservation schemes might place their importance below those areas that are currently entirely neglected. It is, however, possible that there are a higher number of neglected areas for ED species compared to EDGE, since many prioritisation approaches incorporate some measure of threat into their hotspot definitions [5] which would therefore be likely to cover a larger proportion of EDGE species. The importance of this study is, among others, the establishment of a spatial perspective for an otherwise species-centred conservation initiative: the EDGE of Existence programme. These ED and EDGE zones are characterised by an overdispersion of cumulative ED and EDGE scores, including those species in most urgent need of conservation. In the future, it will be important to integrate ED and EDGE zones in the network of existing conservation areas (see also [50]).

## Supporting Information

Table S1 **Sizes and overlap of ED and EDGE zones between the taxa.** Units are km^2^ for the area of the zones.
(CSV)

Table S2 **Model estimates and test statistics for all environmental correlates (PCA 1 to PCA 8) predicting qED and qEDGE for all resolutions and both taxa.**
(CSV)

Table S3 **Model estimates and test statistics for the effect of global land cover types on qED and qEDEG (GLC categories sensu Globcover 2009 V2.3) for all resolutions and both taxa, as well as ED and EDGE.**
(CSV)

Data S1 **Zip compressed species level data of ED and EDGE scores for all mammals and amphibian species included in the analyses.**
(ZIP)

Data S2 **Spatial polygons containing the EDGE and ED zones for amphibians and mammals in a RData file which can be used in R (with the sp package loaded).** Three variables define the different polygons: “Res” refers to the resolution of the rasters used to create the polygons and takes values from 25 to 200 in steps of 25; “Cat” describes whether the polygon represents an ED or EDGE zone; “Taxon” refers to amphibian and mammalian ED and EDGE zones respectively.
(ZIP)

Maps S1 **Species richness maps of the 5% top ranking ED and EDGE amphibian species for resolutions from 25×25 km to 200×200 km in steps of 25 km.**
(PDF)

Maps S2 **Species richness maps of the 5% top ranking ED and EDGE mammalian species for resolutions from 25×25 km to 200×200 km in steps of 25 km.**
(PDF)

Maps S3 **qED and qEDGE maps for amphibians for raster resolutions of 25×25 km to 200×200 km in steps of 25 km.**
(PDF)

Maps S4 **qED and qEDGE maps for mammals for raster resolutions of 25×25 km to 200×200 km in steps of 25 km.**
(PDF)
