# Supplementary material for: Global Patterns of Evolutionary Distinct and Globally Endangered Amphibians and Mammals
Source: PLoS One. 2013 May 15;8(5):e63582. doi: 10.1371/journal.pone.0063582 (PMC3655148; doi:10.1371/journal.pone.0063582)
Supplement: Maps S1 — Species richness maps of the 5% top ranking ED and EDGE amphibian species for resolutions from 25×25 km to 200×200 km in steps of 25 km. (PDF) [file pone.0063582.s006.pdf]

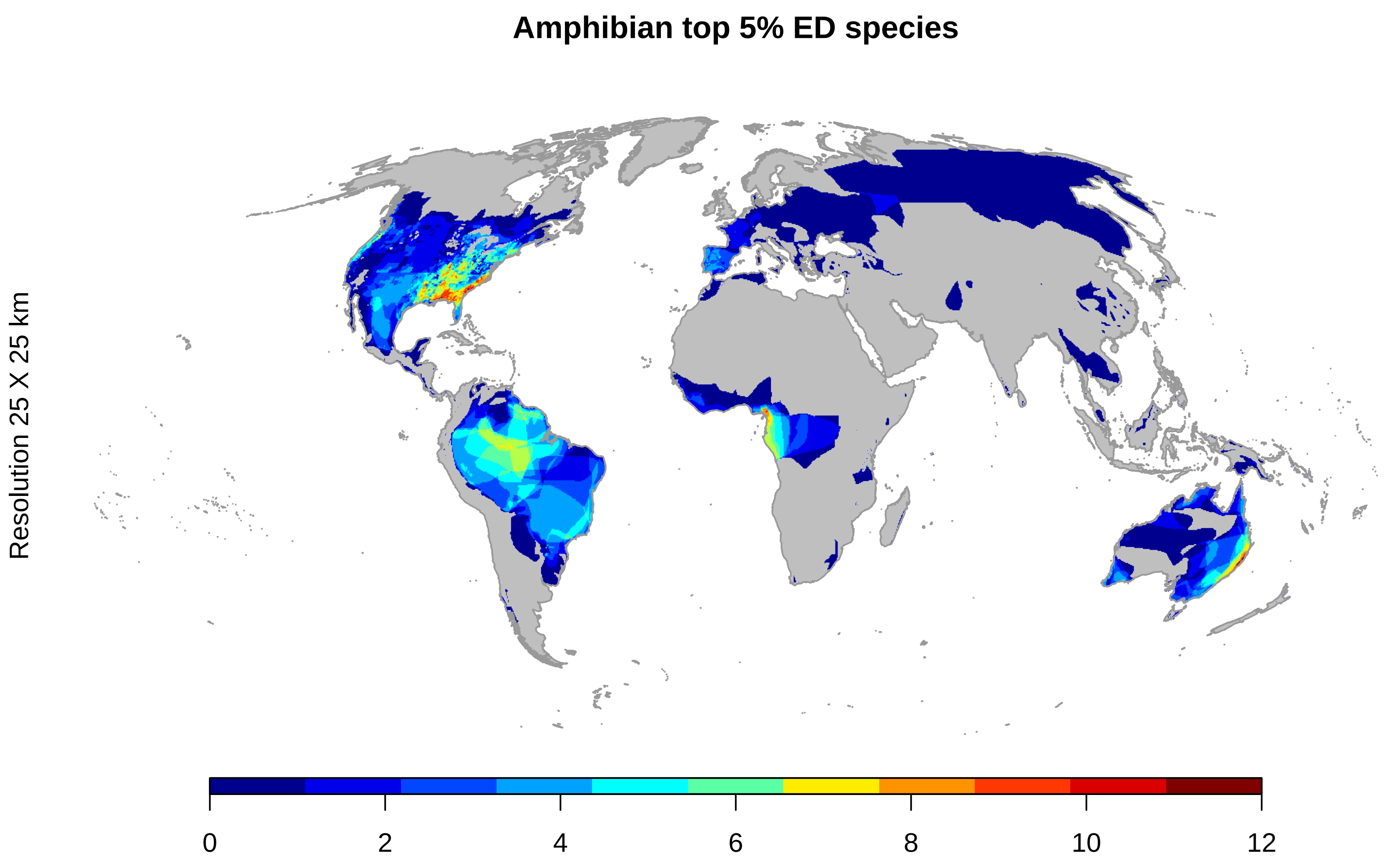
Amphibian top 5% ED species
Resolution 25 X 25 km
0
2
4
6
8
10
12

## Amphibian top 5% EDGE species

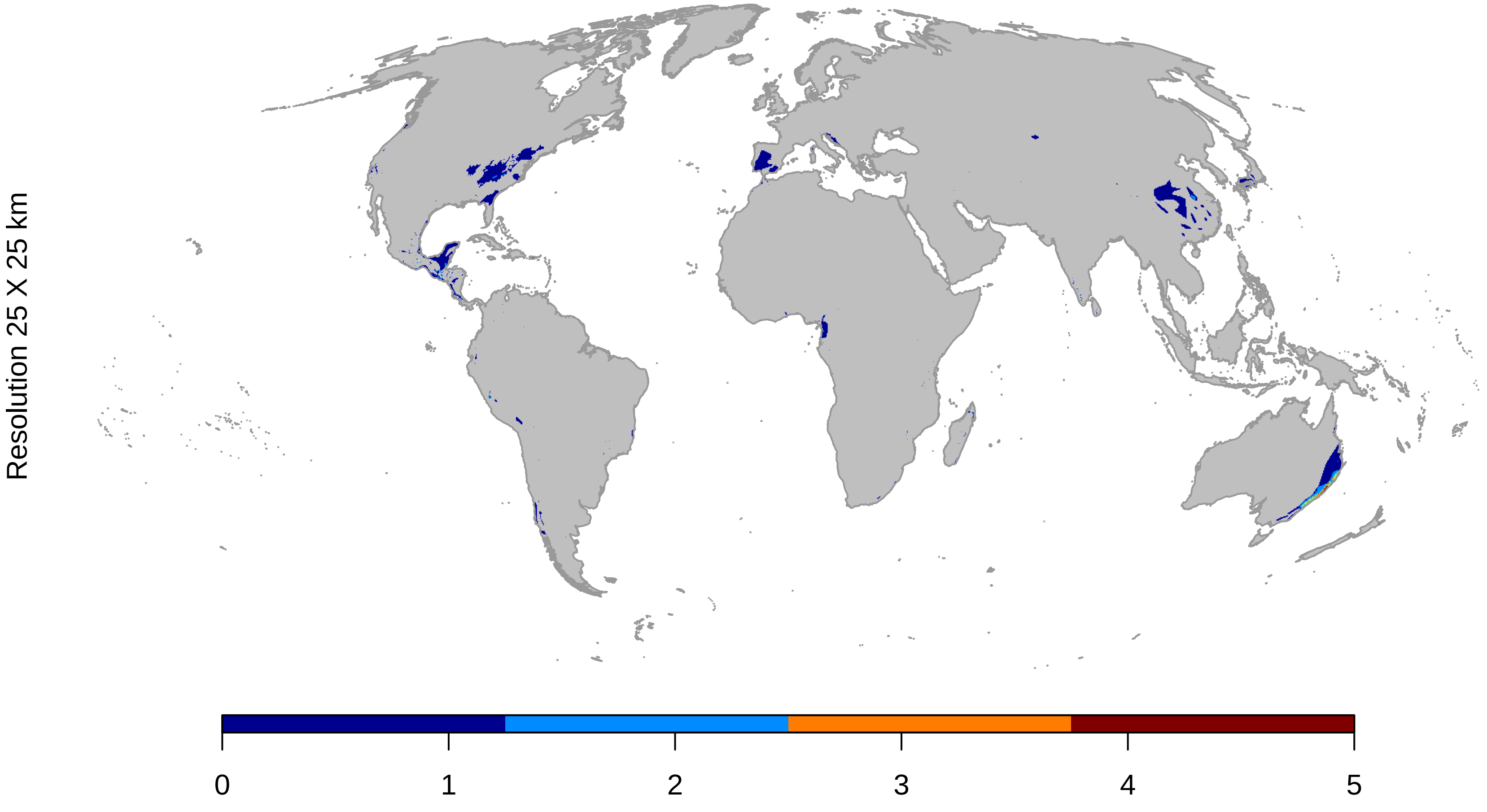

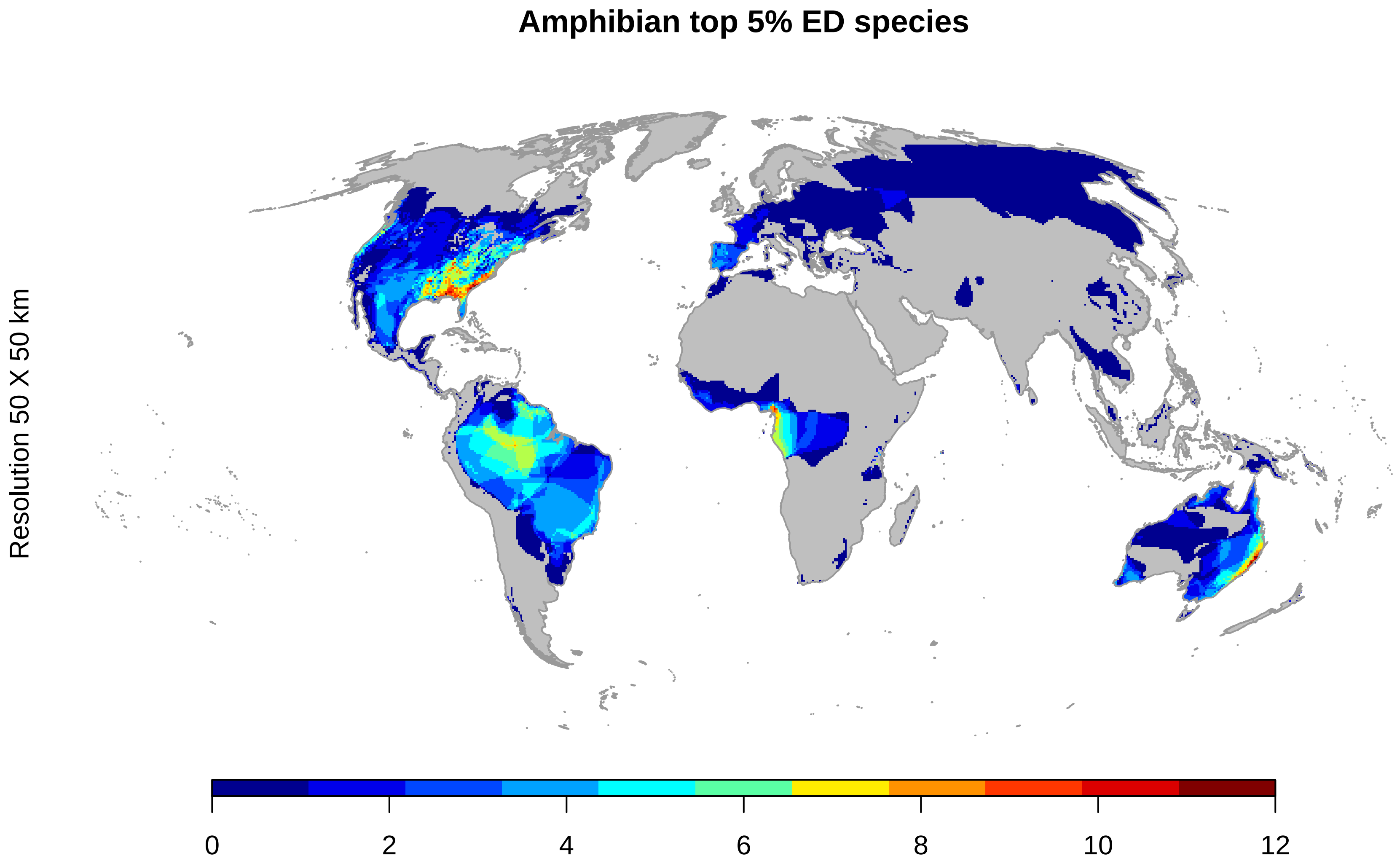

Amphibian top 5% ED species
Resolution 50 X 50 km
0
2
4
6
8
10
12

# Amphibian top 5% EDGE species

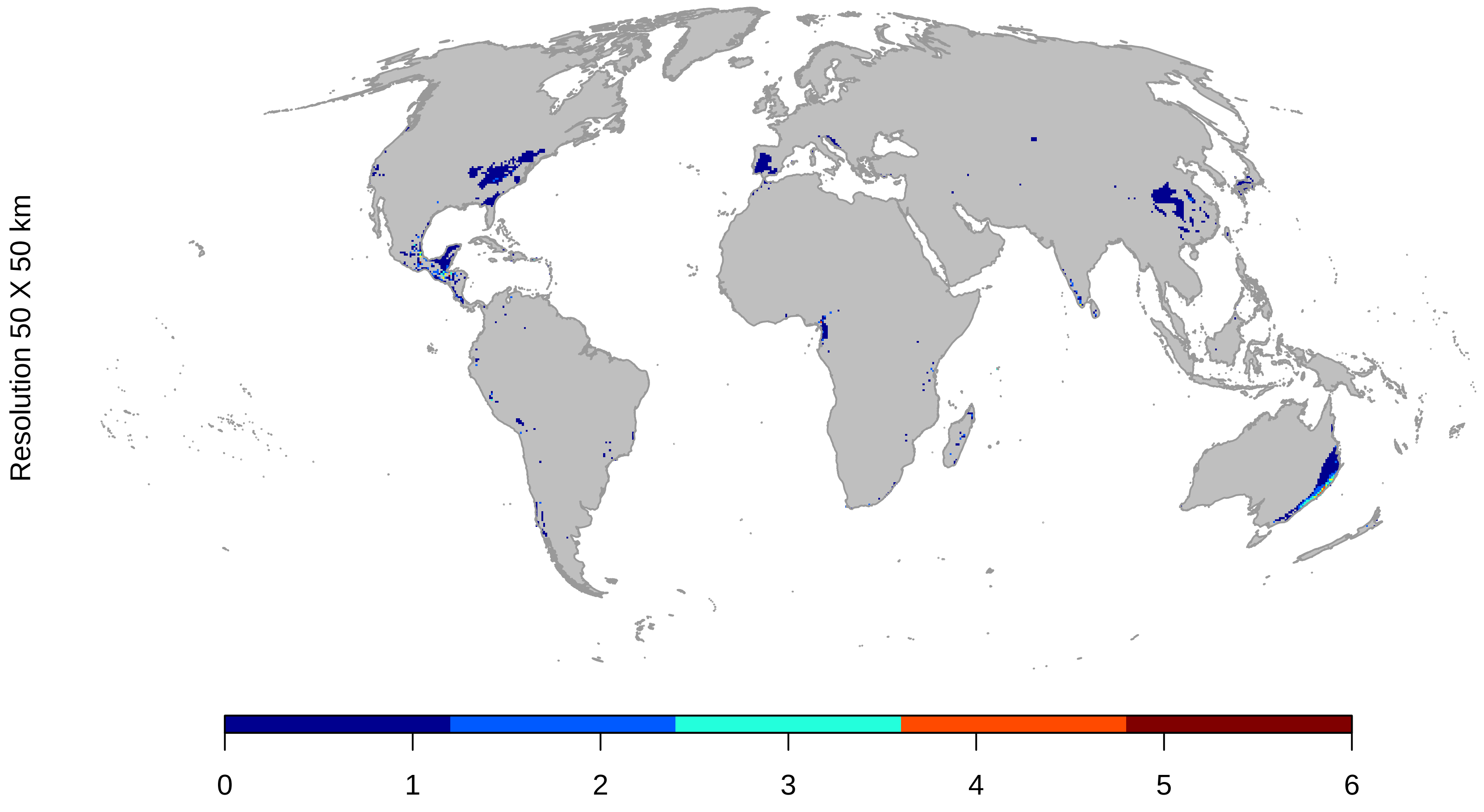

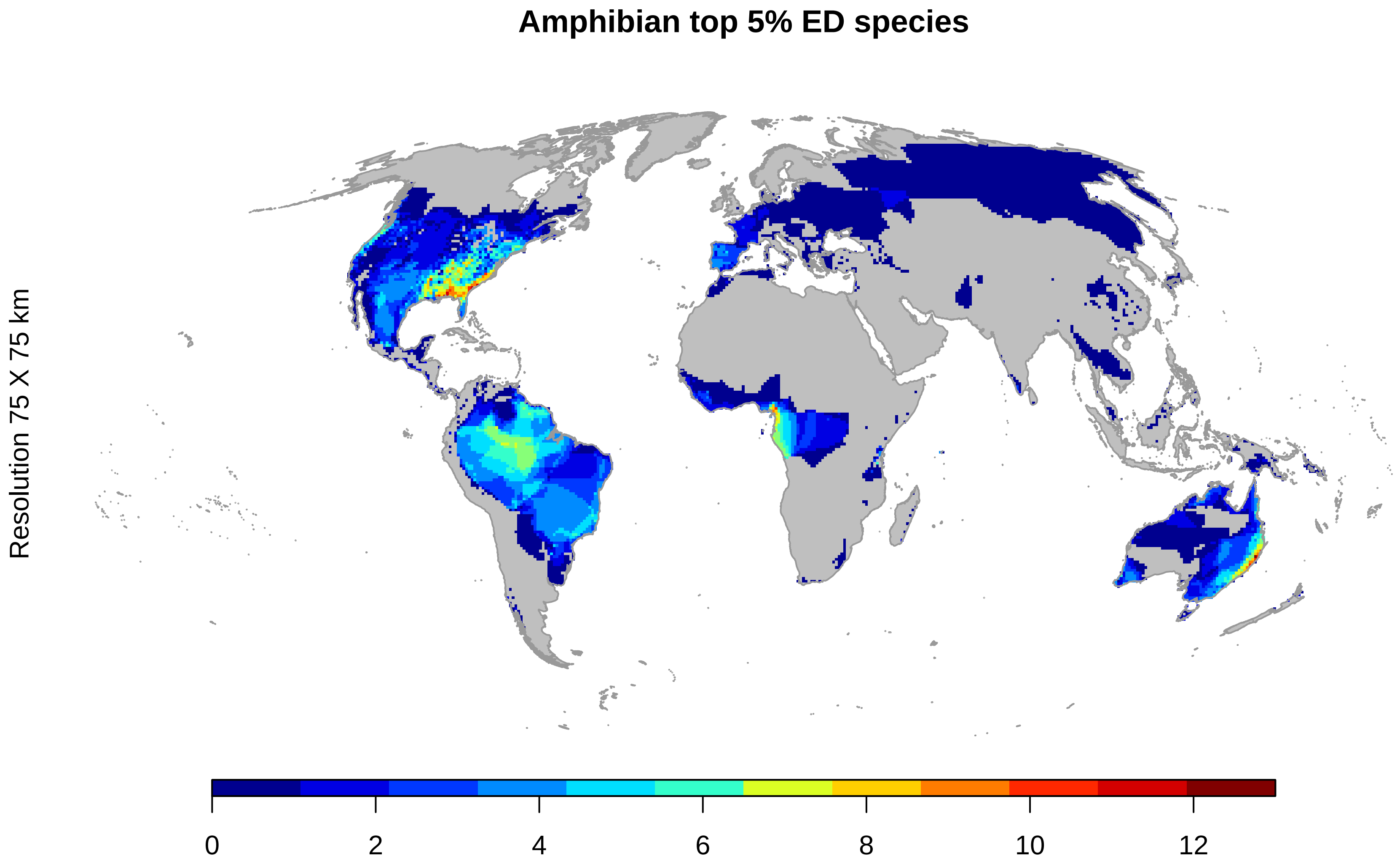
Amphibian top 5% ED species
Resolution 75 X 75 km
0
2
4
6
8
10
12

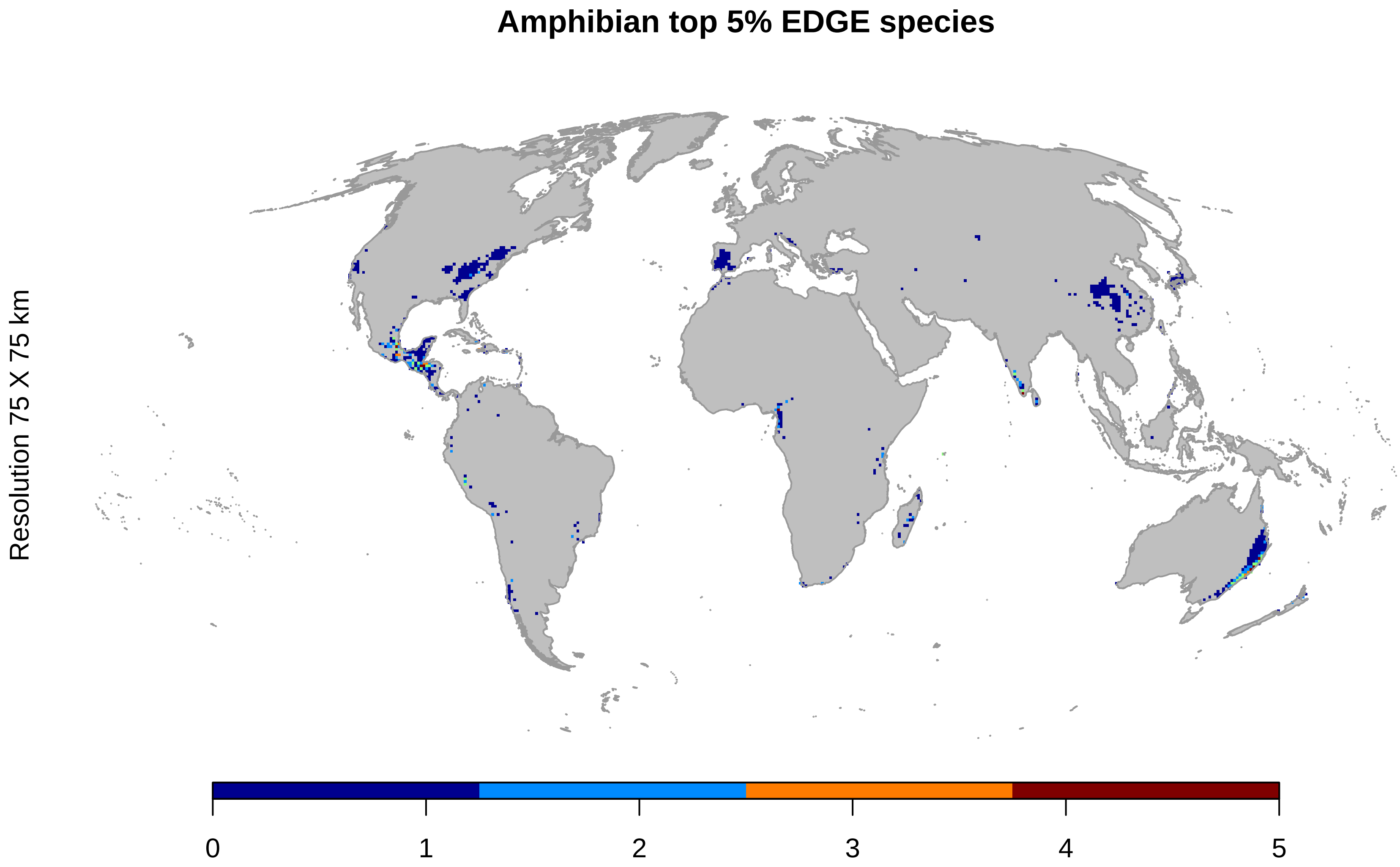

Amphibian top 5% EDGE species
Resolution 75 X 75 km
0
1
2
3
4
5

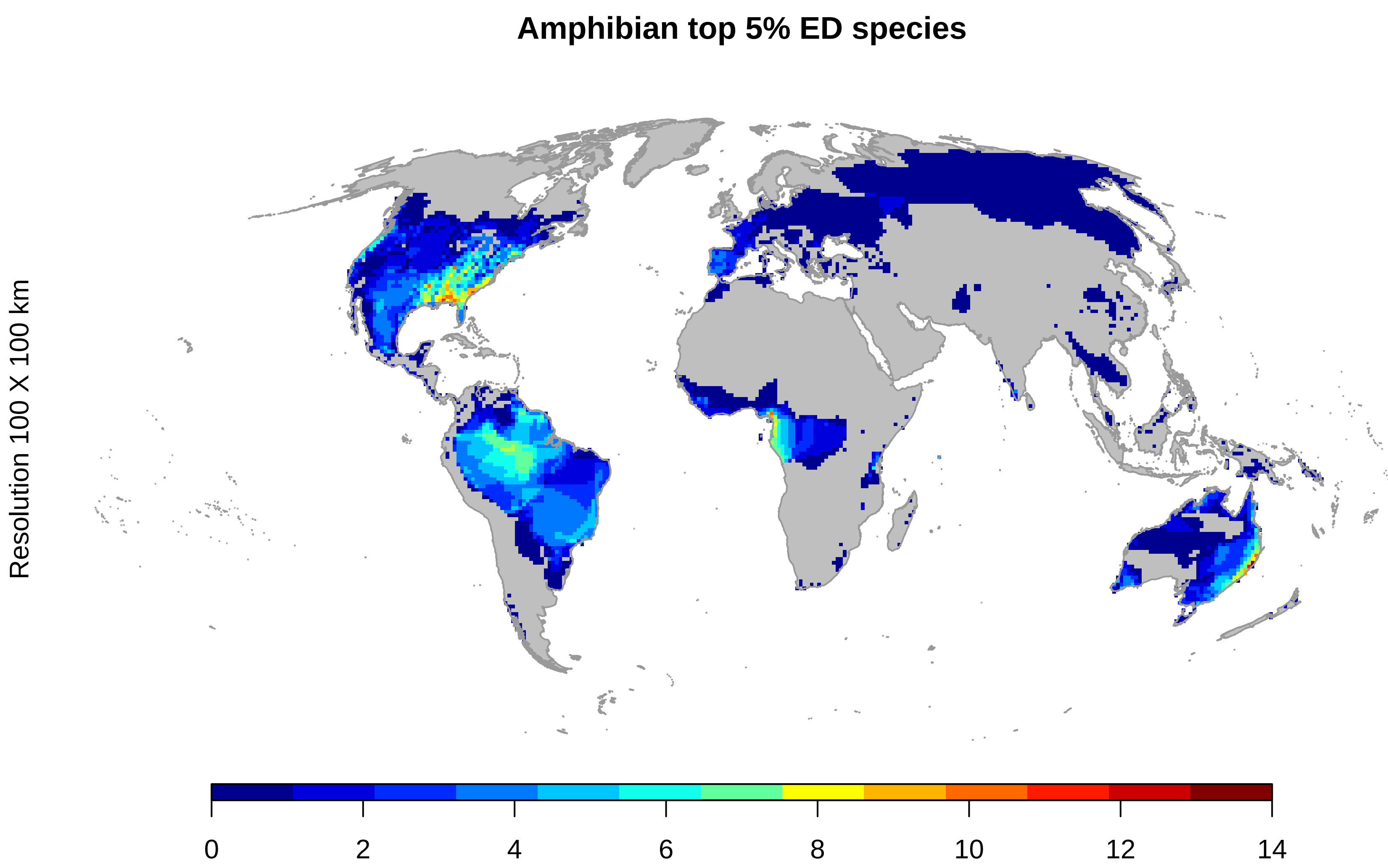
Amphibian top 5% ED species
Resolution 100 X 100 km
0
2
4
6
8
10
12
14

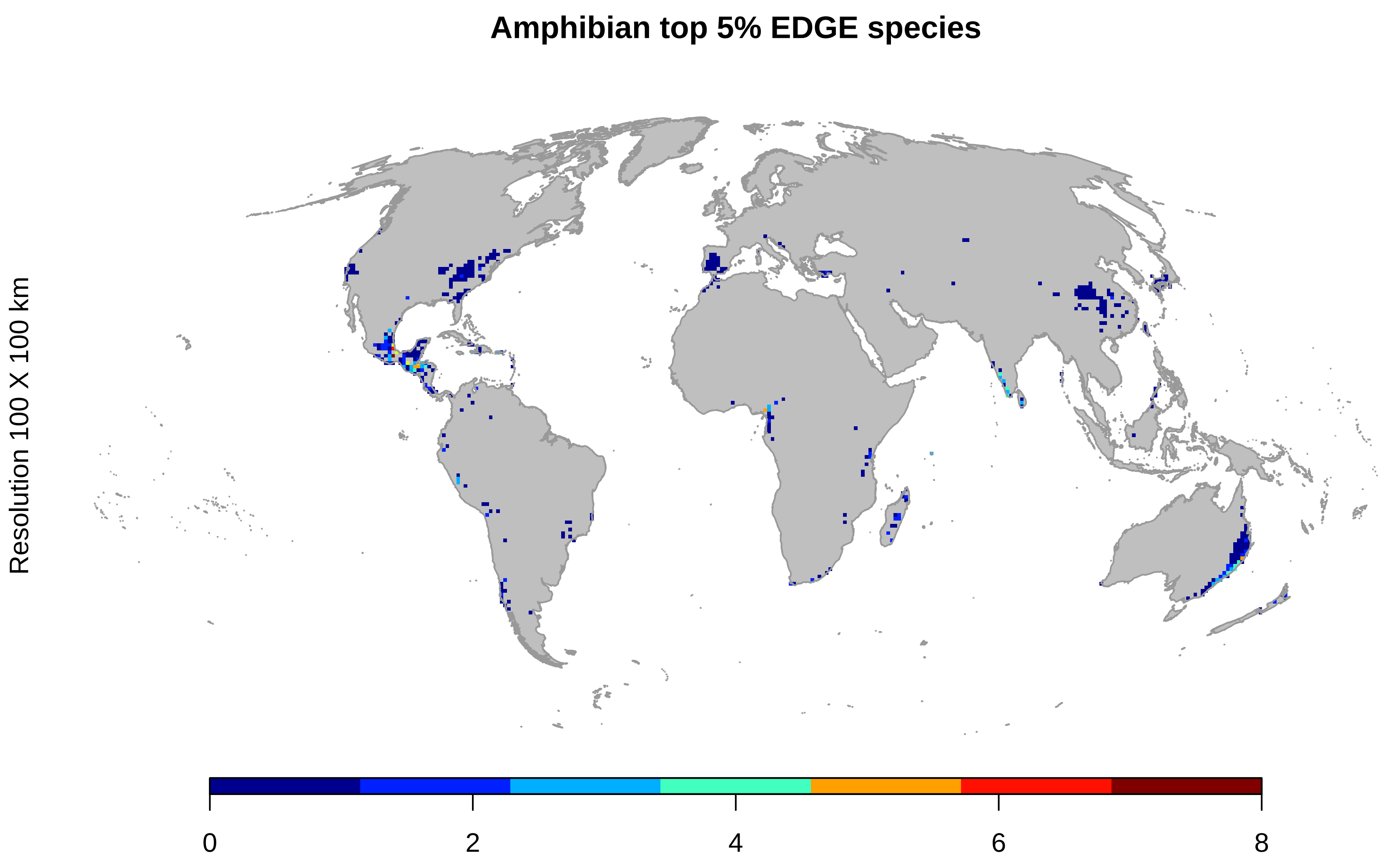
Amphibian top 5% EDGE species
Resolution 100 X 100 km
0
2
4
6
8

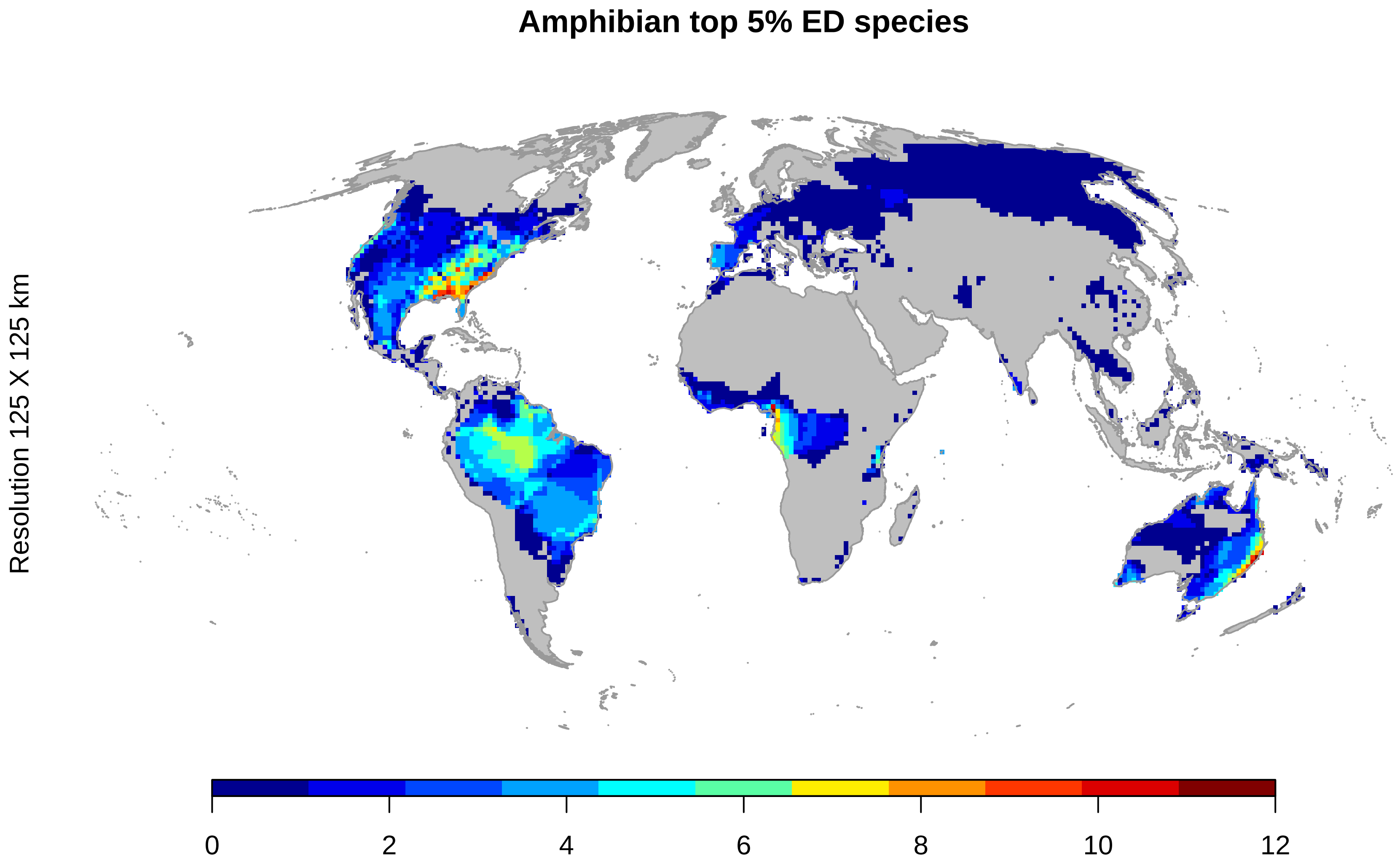

Amphibian top 5% ED species
Resolution 125 X 125 km
0
2
4
6
8
10
12

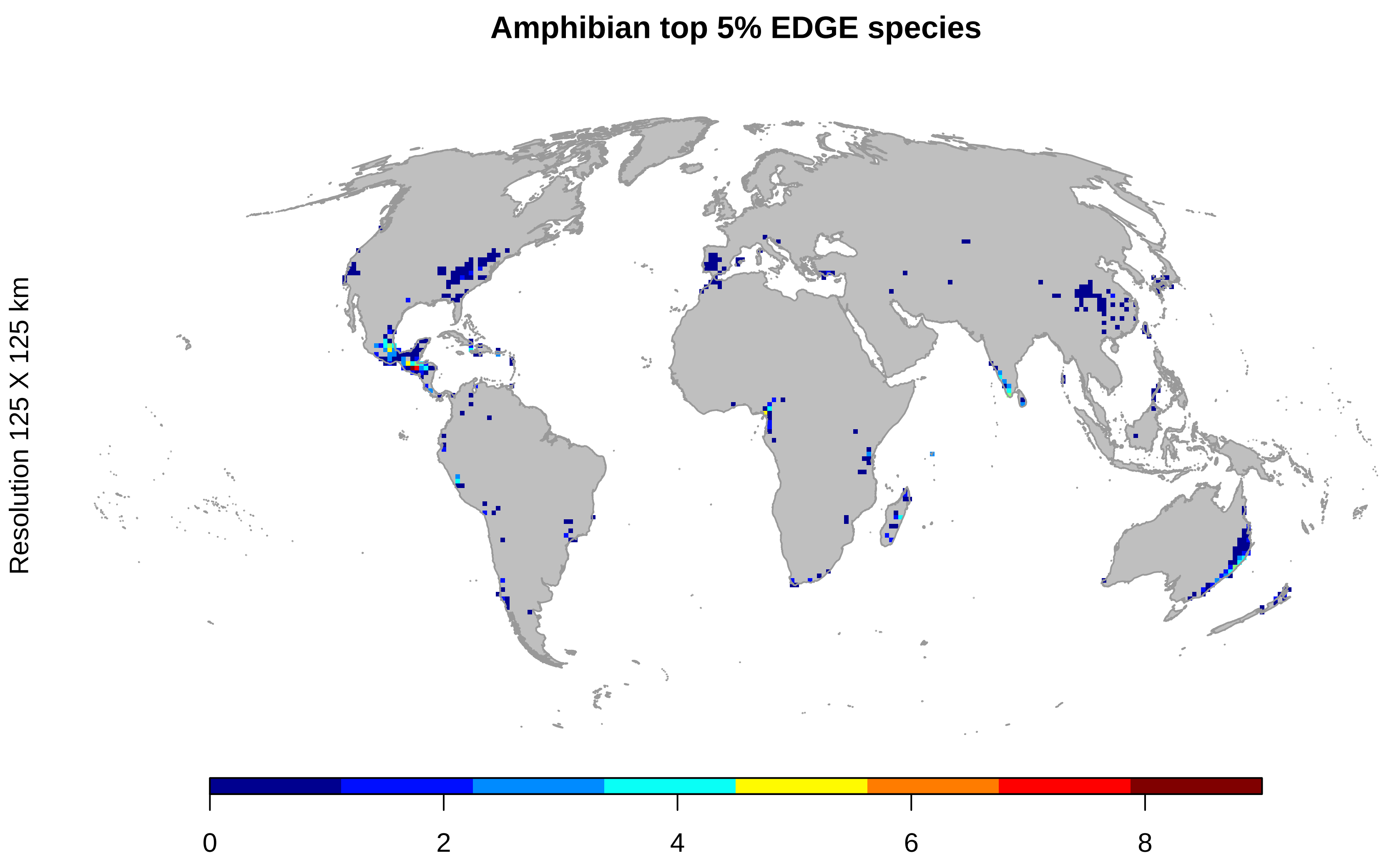

Amphibian top 5% EDGE species
Resolution 125 X 125 km
0
2
4
6
8

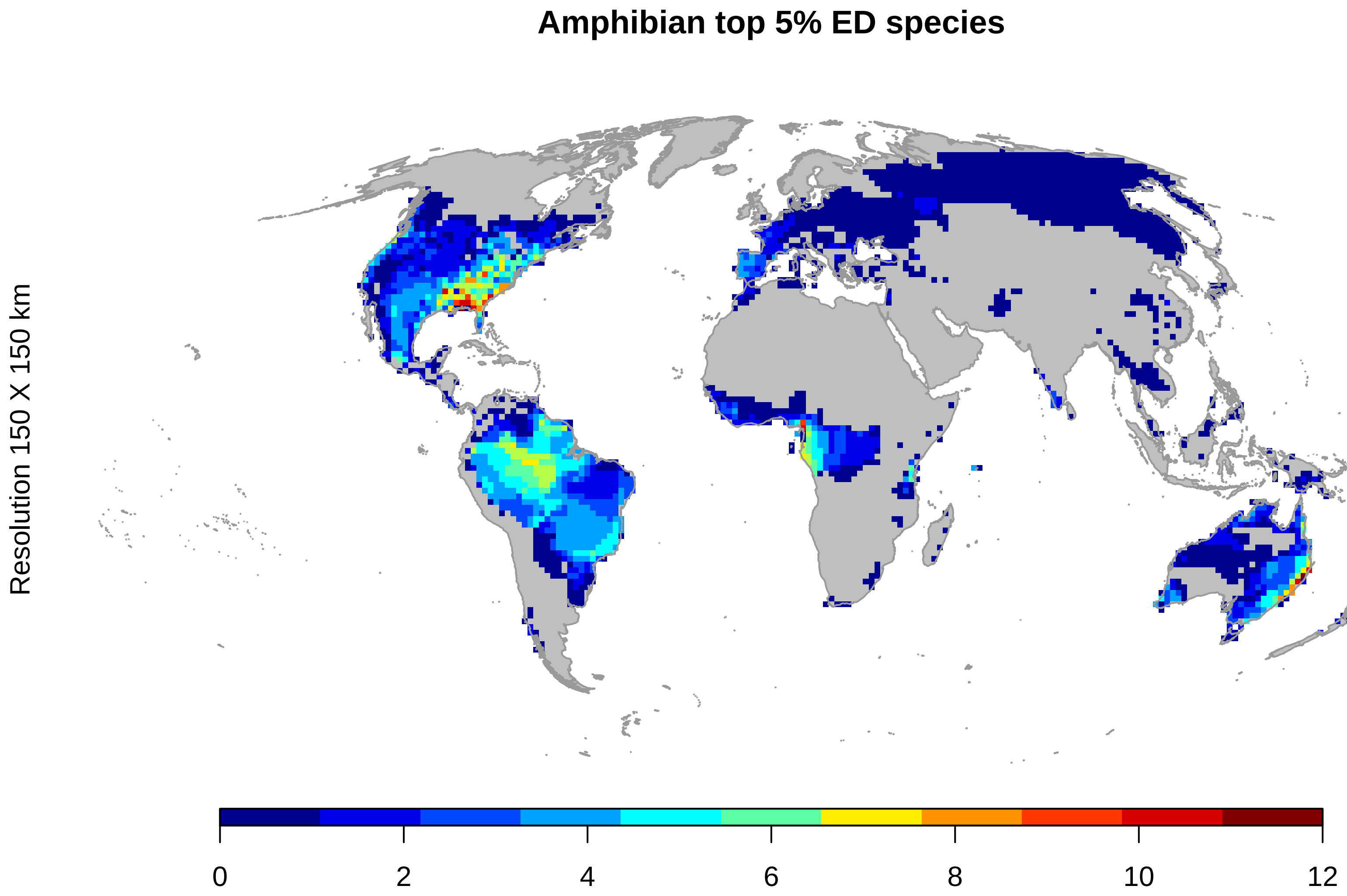
Amphibian top 5% ED species
Resolution 150 X 150 km
0
2
4
6
8
10
12

# Amphibian top 5% EDGE species

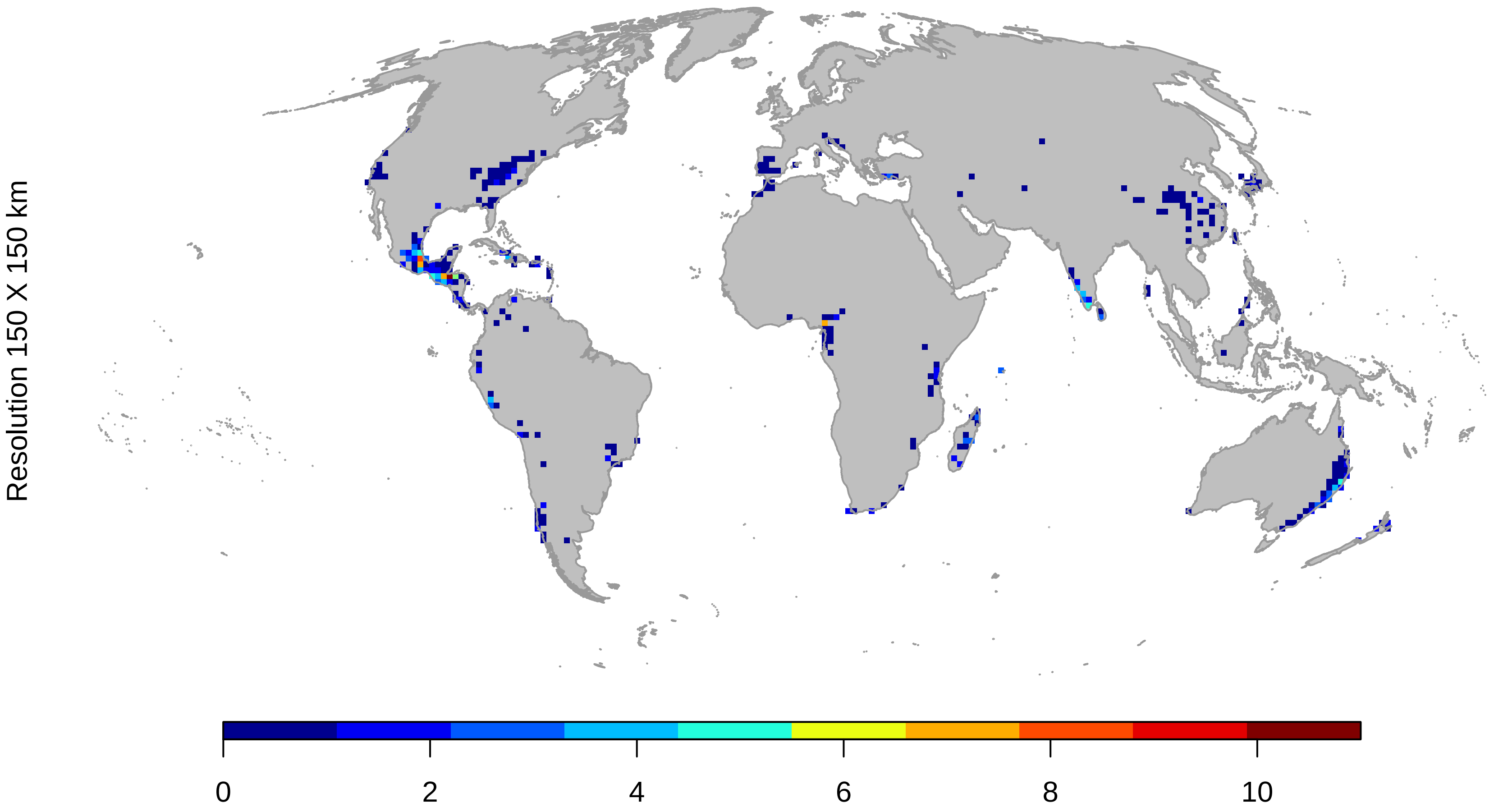

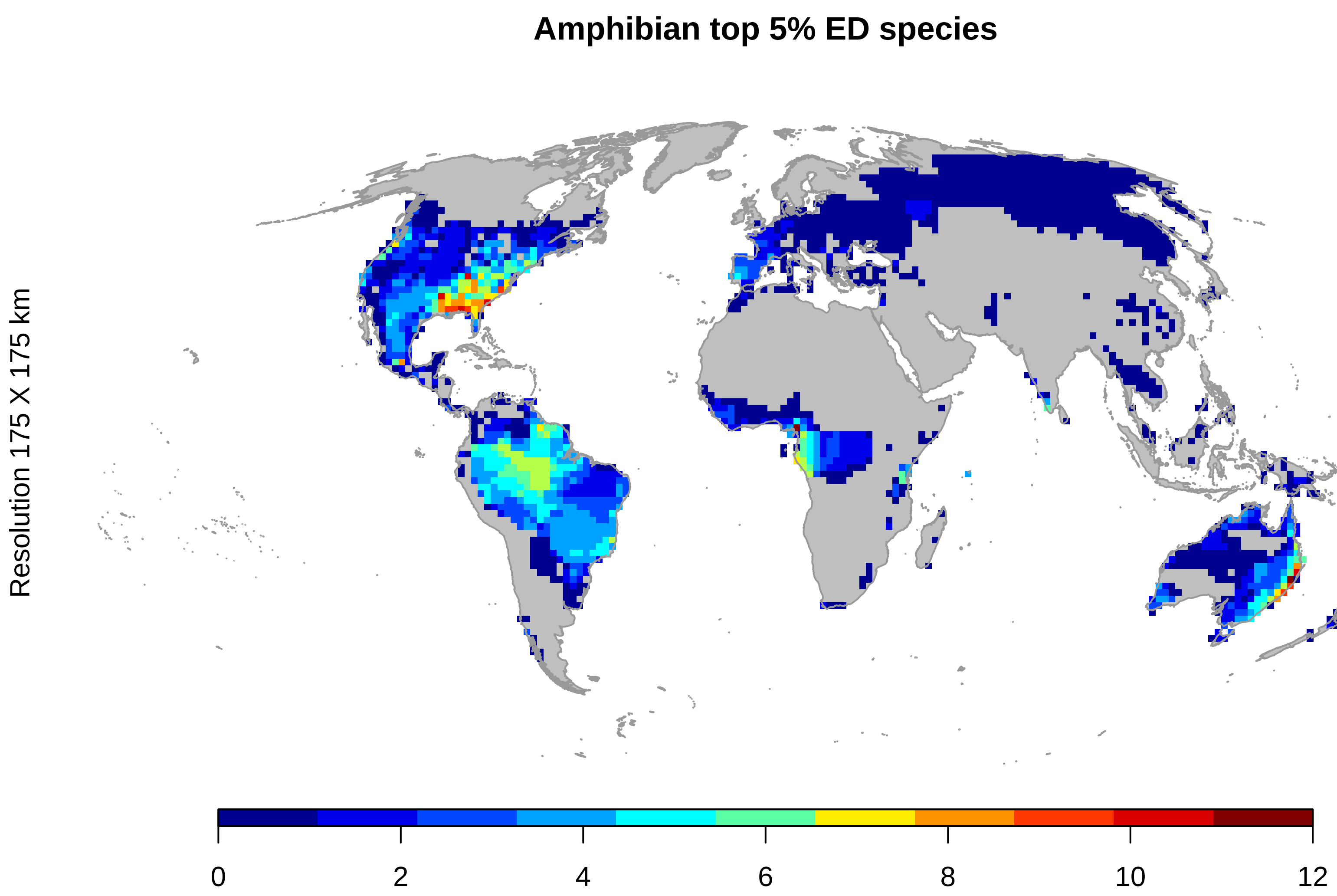
Amphibian top 5% ED species
Resolution 175 X 175 km
0
2
4
6
8
10
12

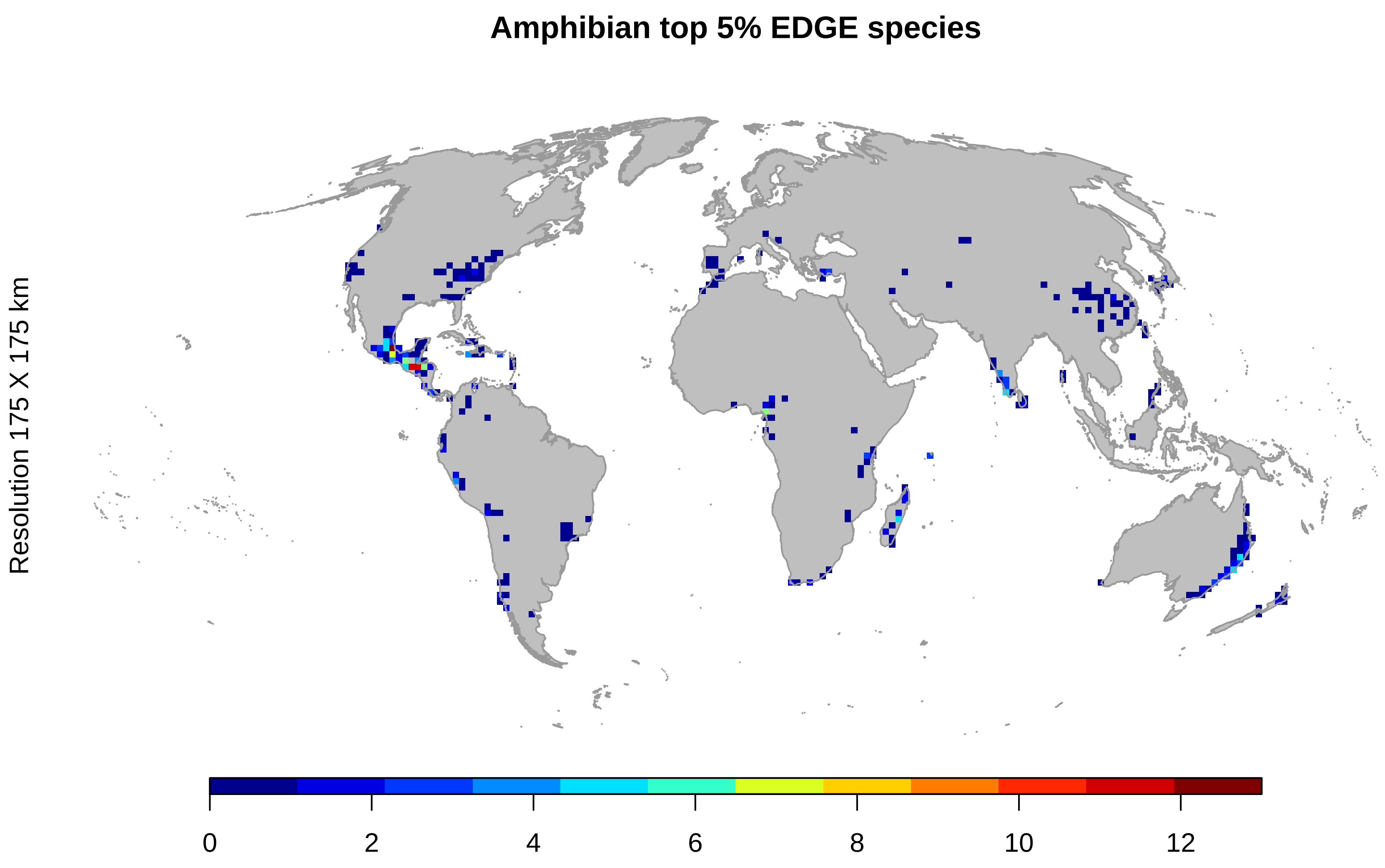

Amphibian top 5% EDGE species
Resolution 175 X 175 km
0
2
4
6
8
10
12

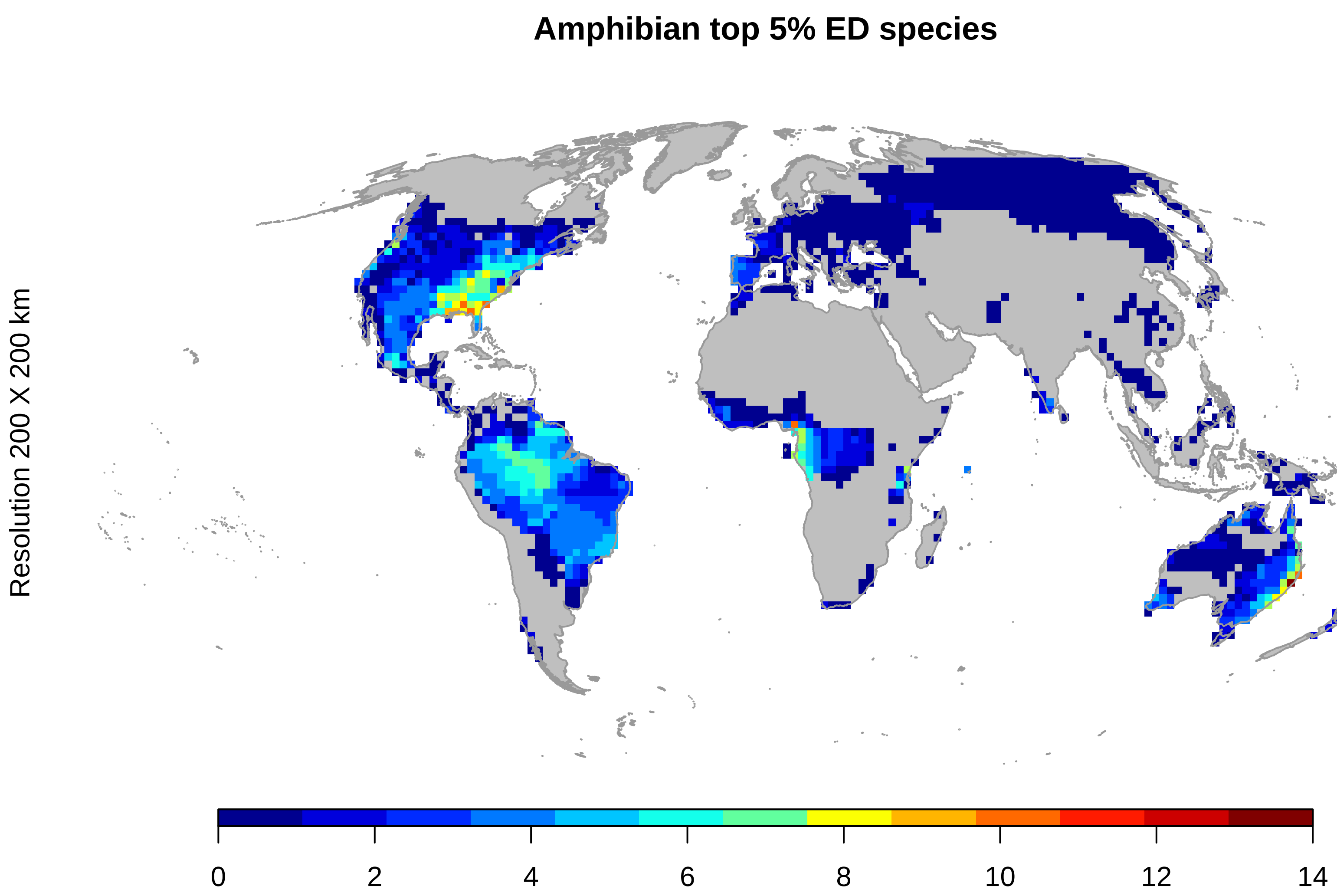
Amphibian top 5% ED species
Resolution 200 X 200 km
0
2
4
6
8
10
12
14

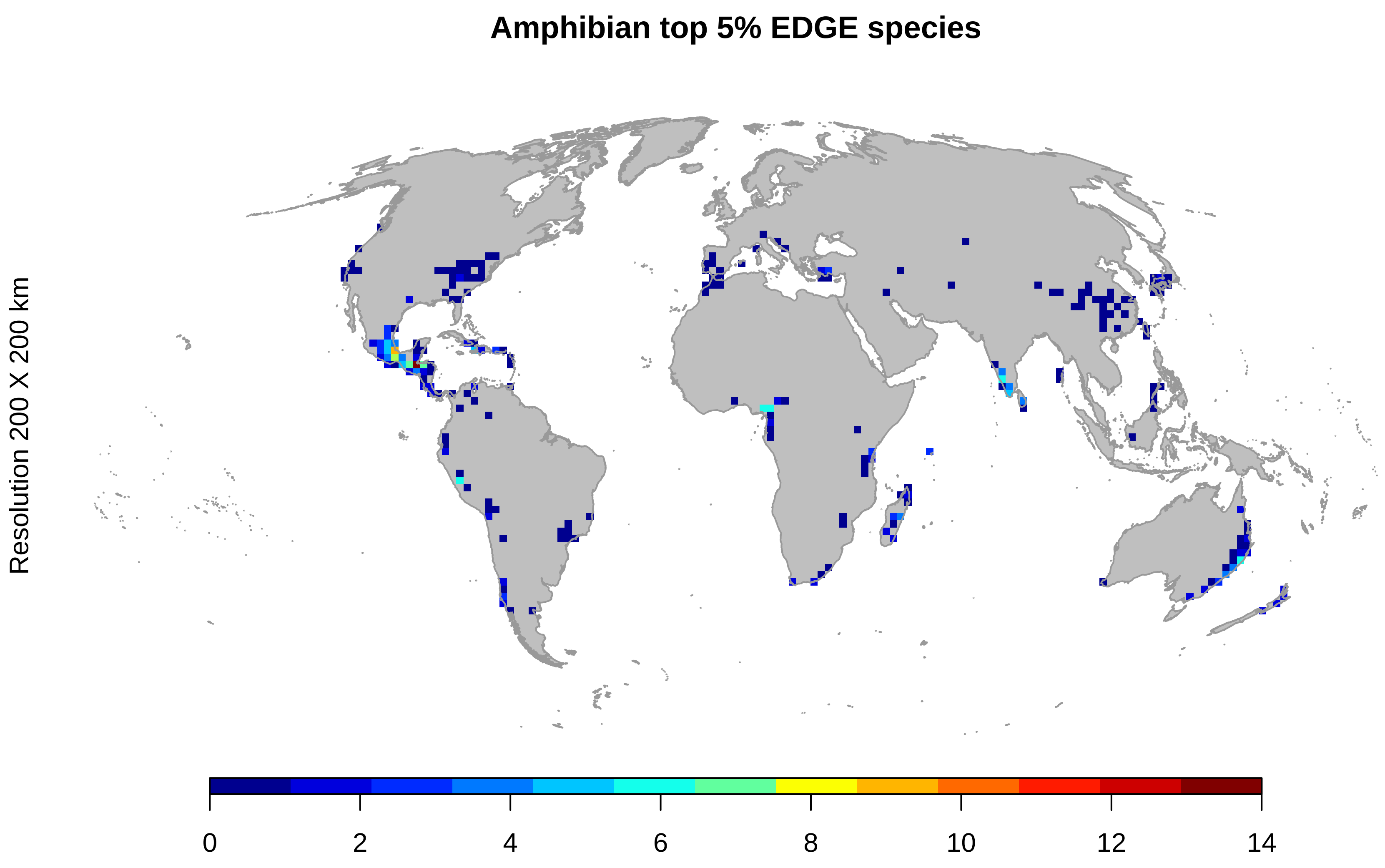
Amphibian top 5% EDGE species
Resolution 200 X 200 km
0
2
4
6
8
10
12
14
