# Supplementary material for: Global Patterns of Evolutionary Distinct and Globally Endangered Amphibians and Mammals
Source: PLoS One. 2013 May 15;8(5):e63582. doi: 10.1371/journal.pone.0063582 (PMC3655148; doi:10.1371/journal.pone.0063582)

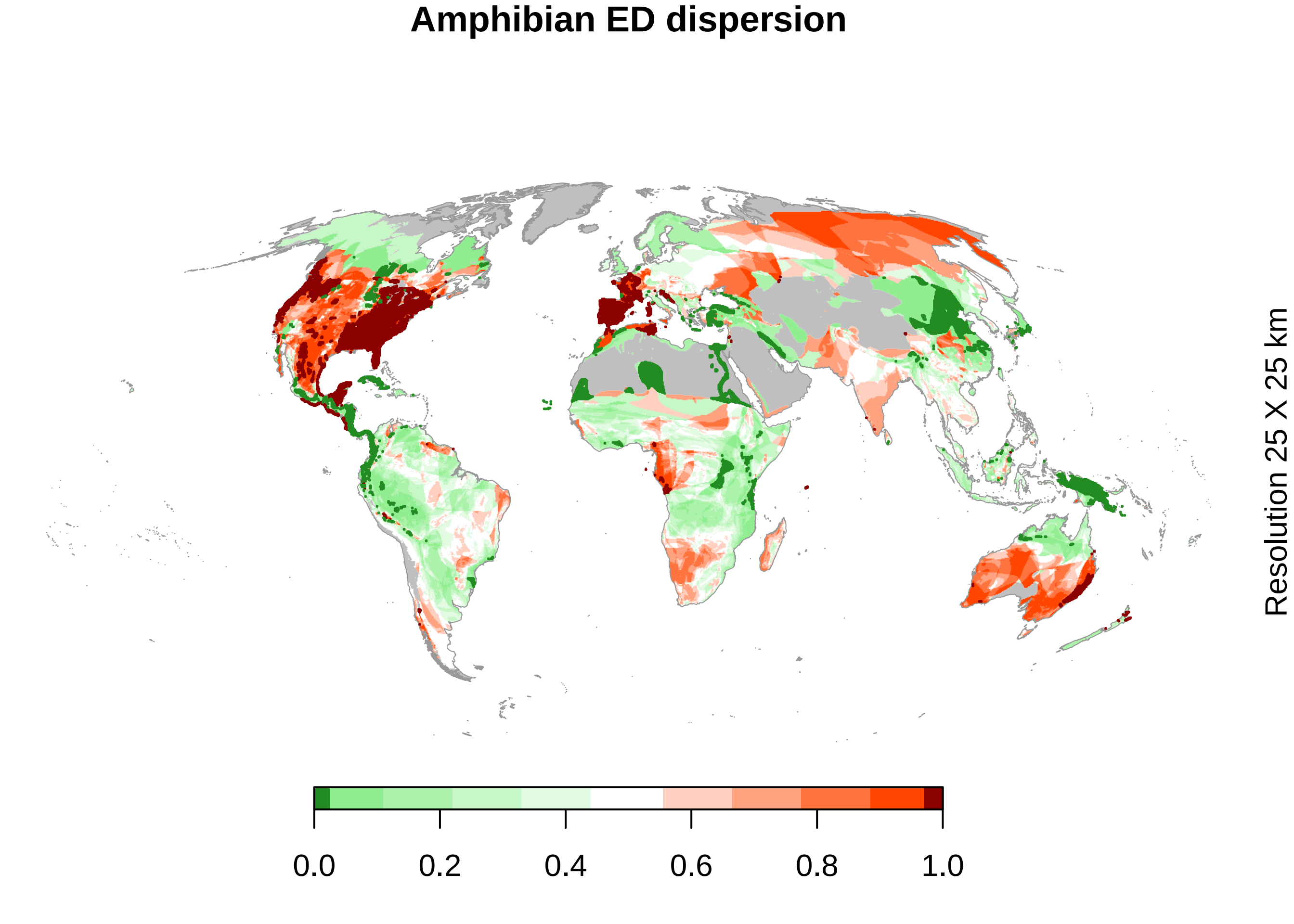
Amphibian ED dispersion
Resolution 25 X 25 km
0.0
0.2
0.4
0.6
0.8
1.0

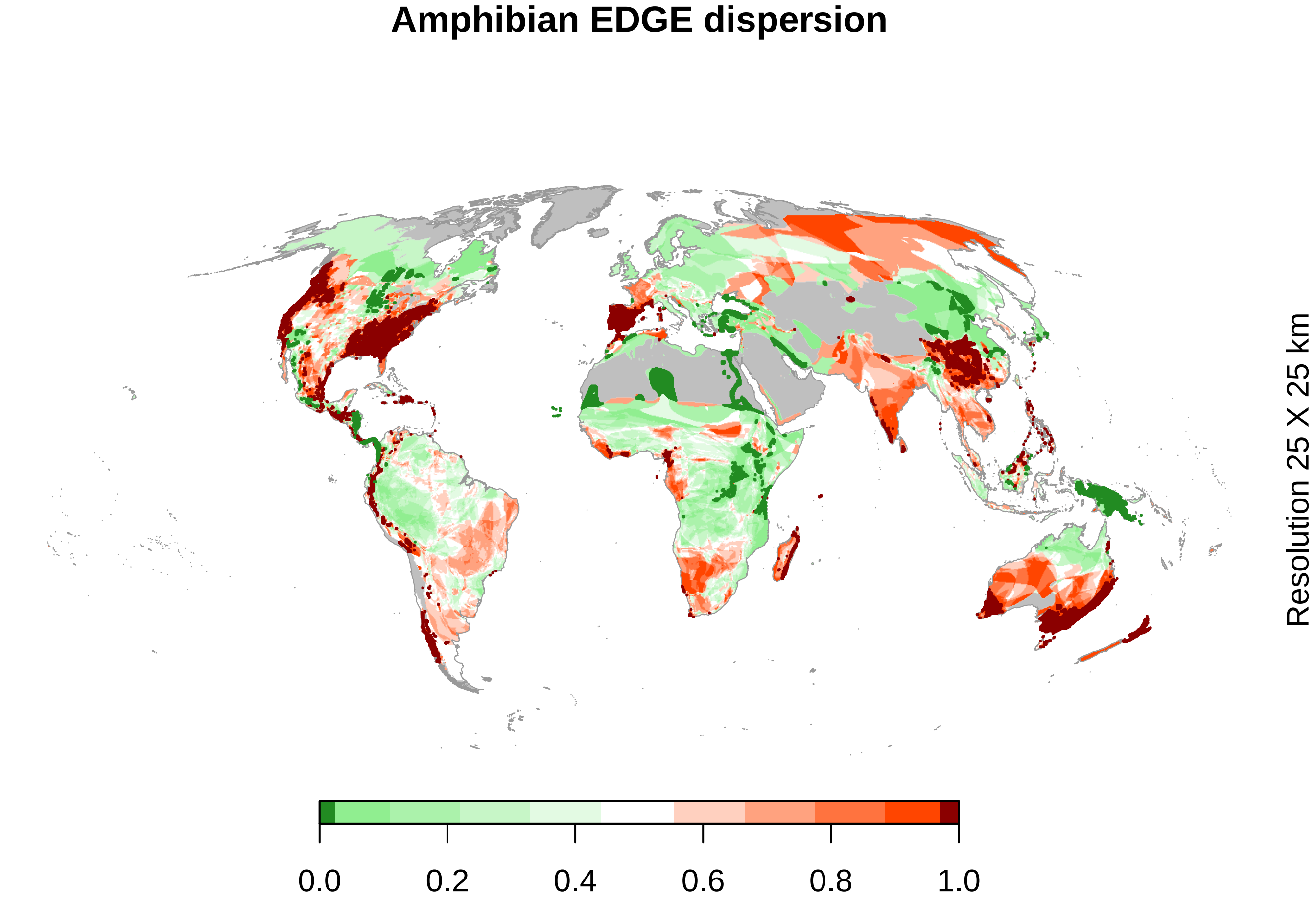

Amphibian EDGE dispersion
Resolution 25 X 25 km
0.0
0.2
0.4
0.6
0.8
1.0

Amphibian ED dispersion

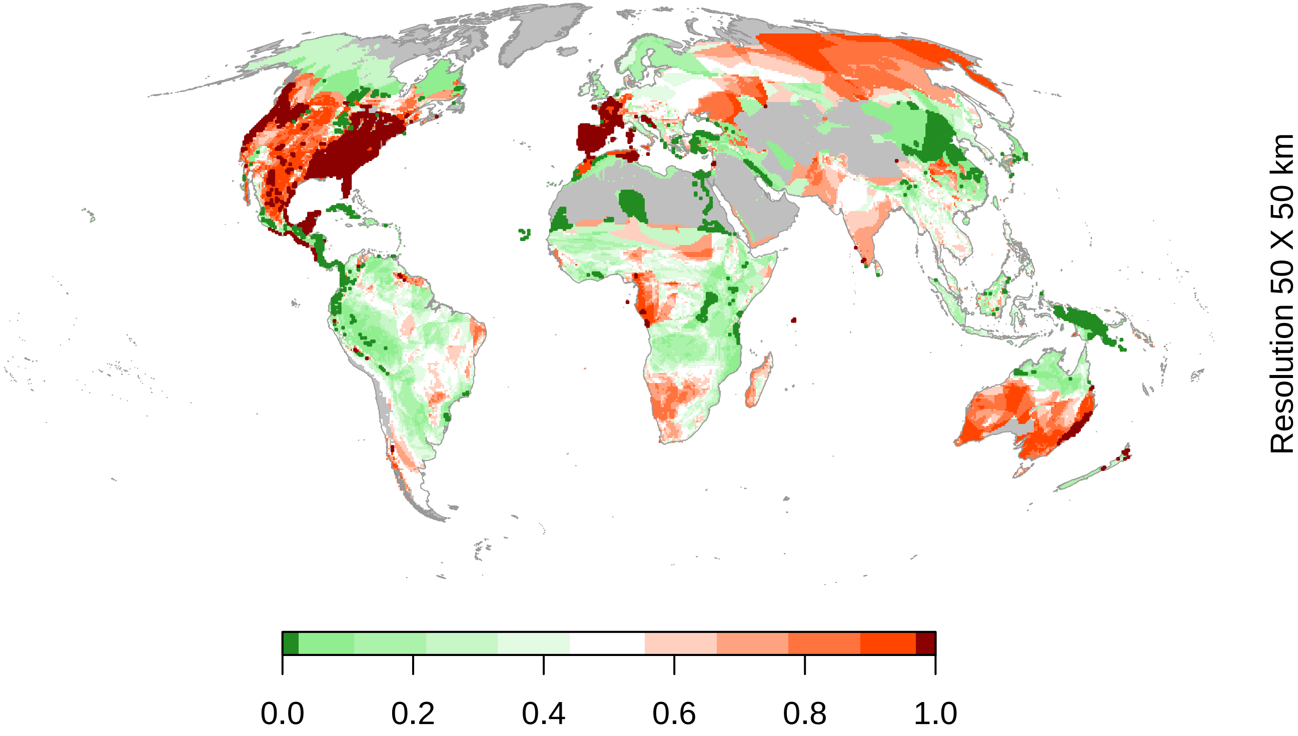

Amphibian EDGE dispersion

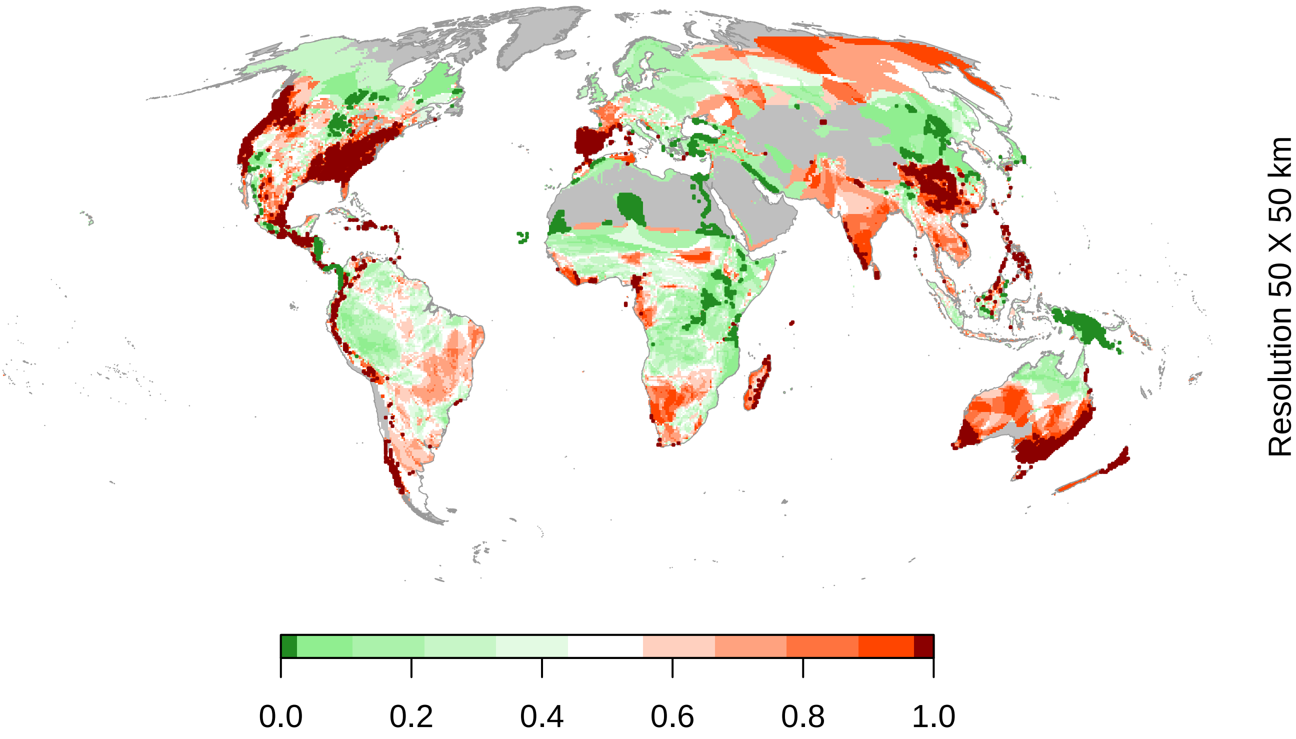

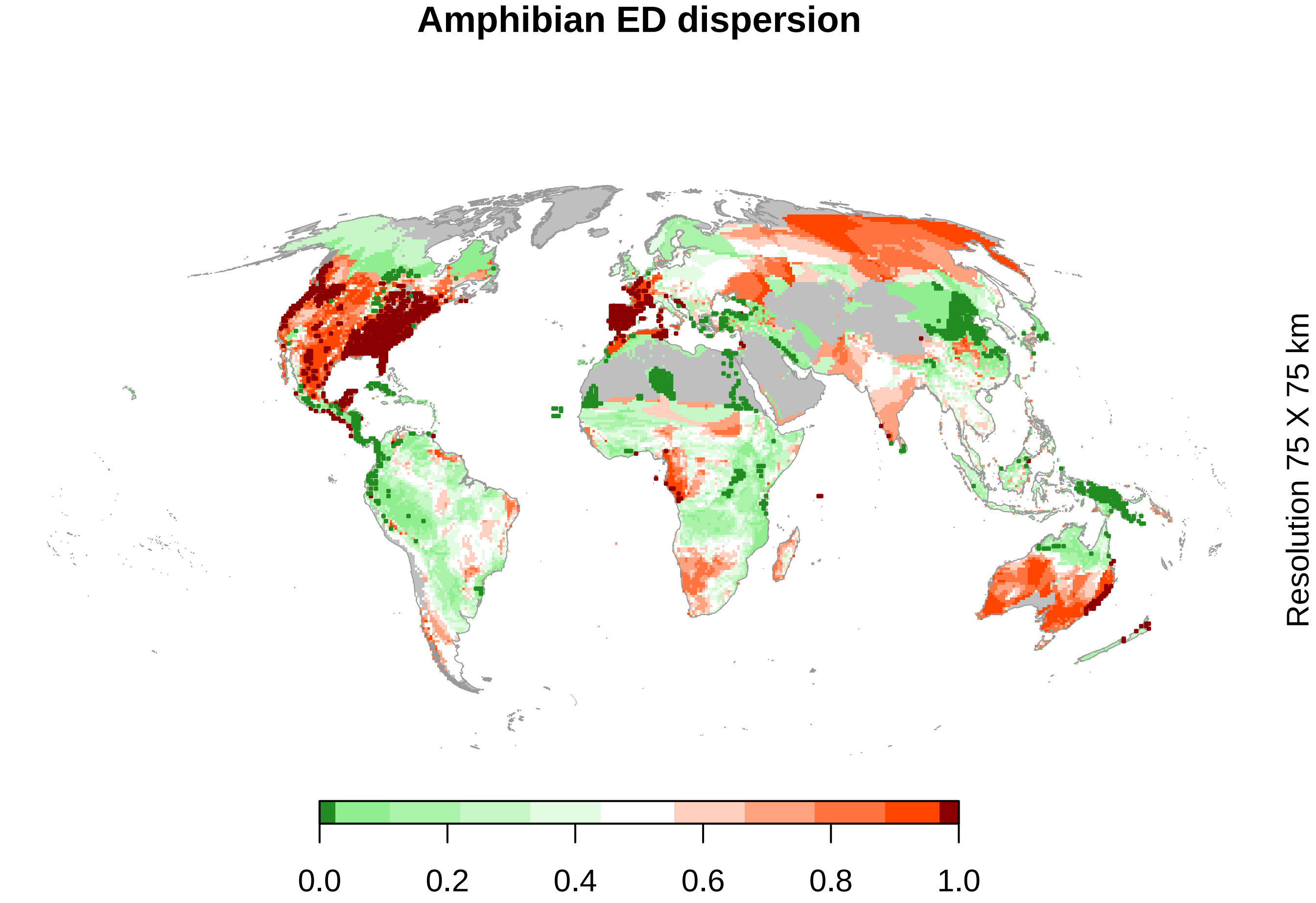
Amphibian ED dispersion
0.0
0.2
0.4
0.6
0.8
1.0
Resolution 75 X 75 km

Amphibian EDGE dispersion

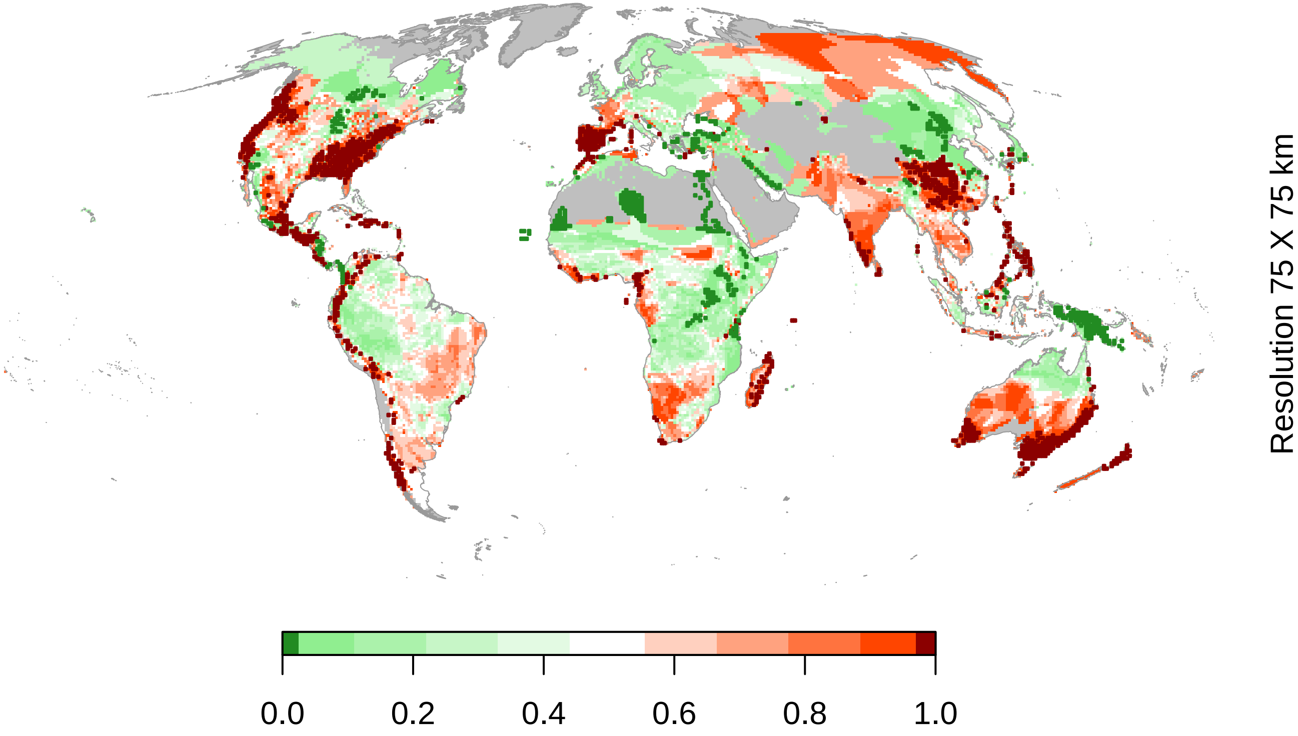

Amphibian ED dispersion

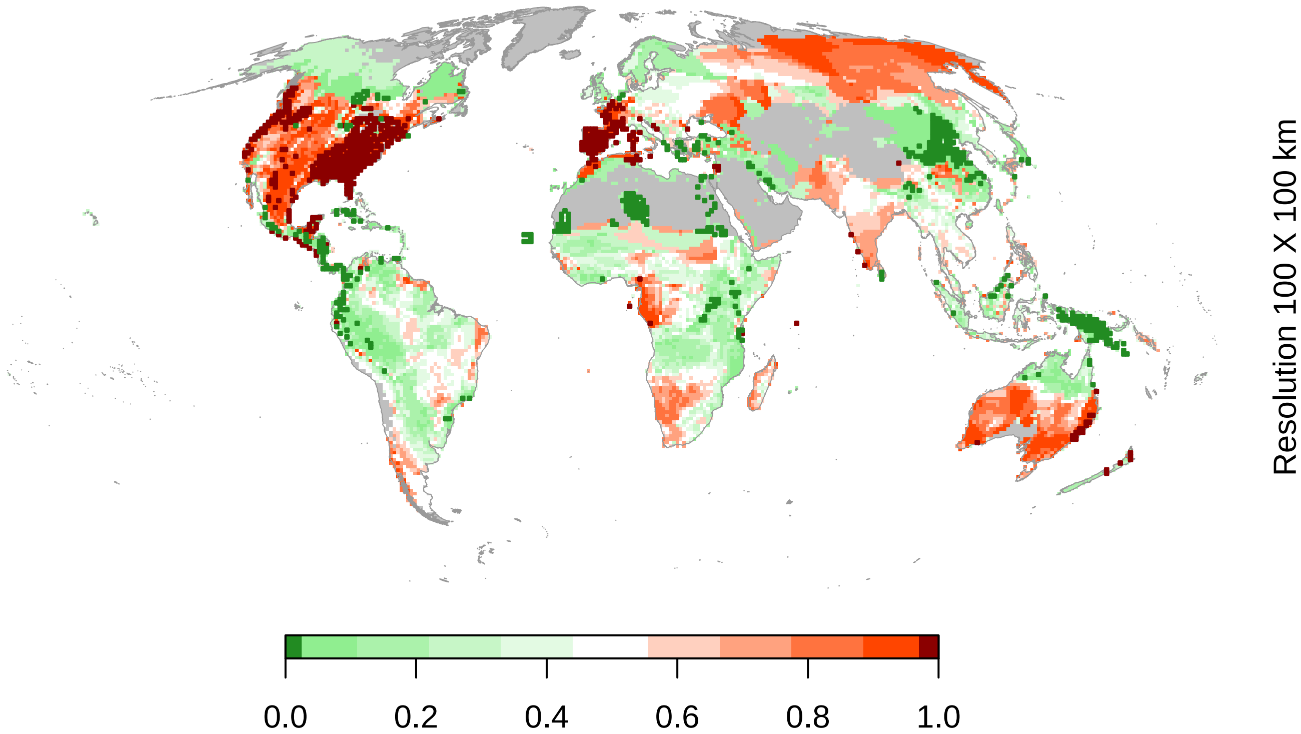

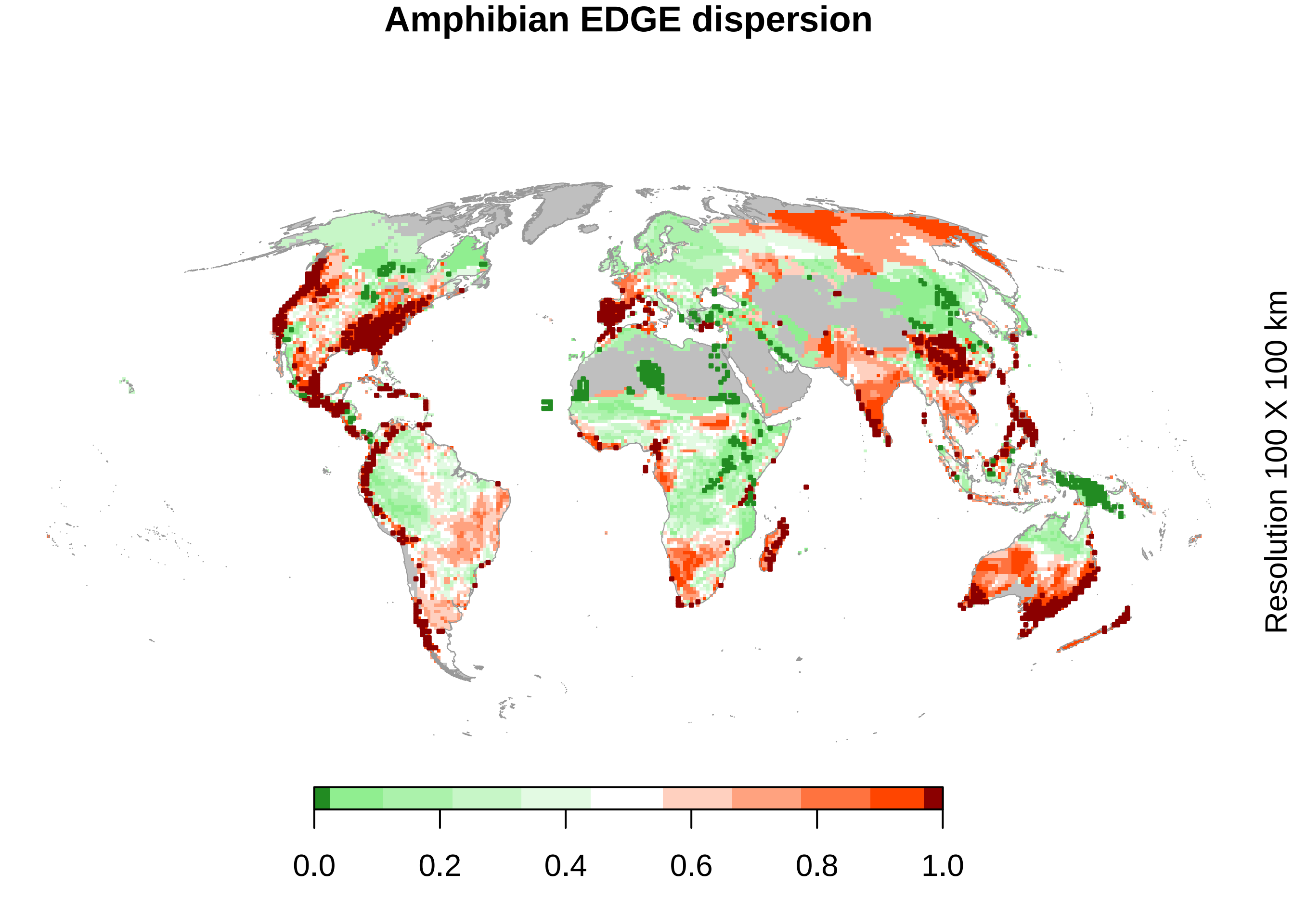
Amphibian EDGE dispersion
Resolution 100 X 100 km
0.0
0.2
0.4
0.6
0.8
1.0

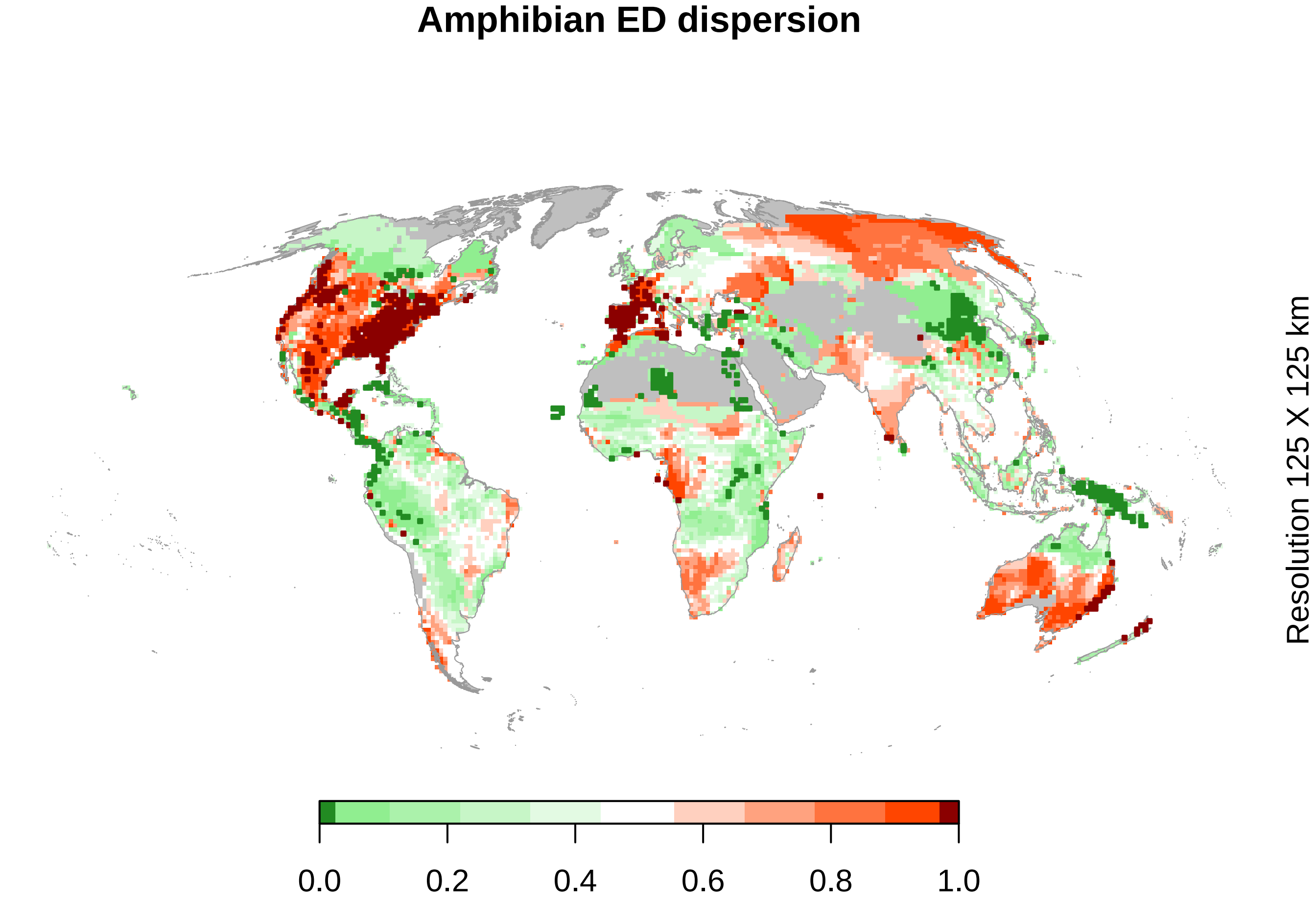
Amphibian ED dispersion
Resolution 125 X 125 km
0.0
0.2
0.4
0.6
0.8
1.0

Amphibian EDGE dispersion

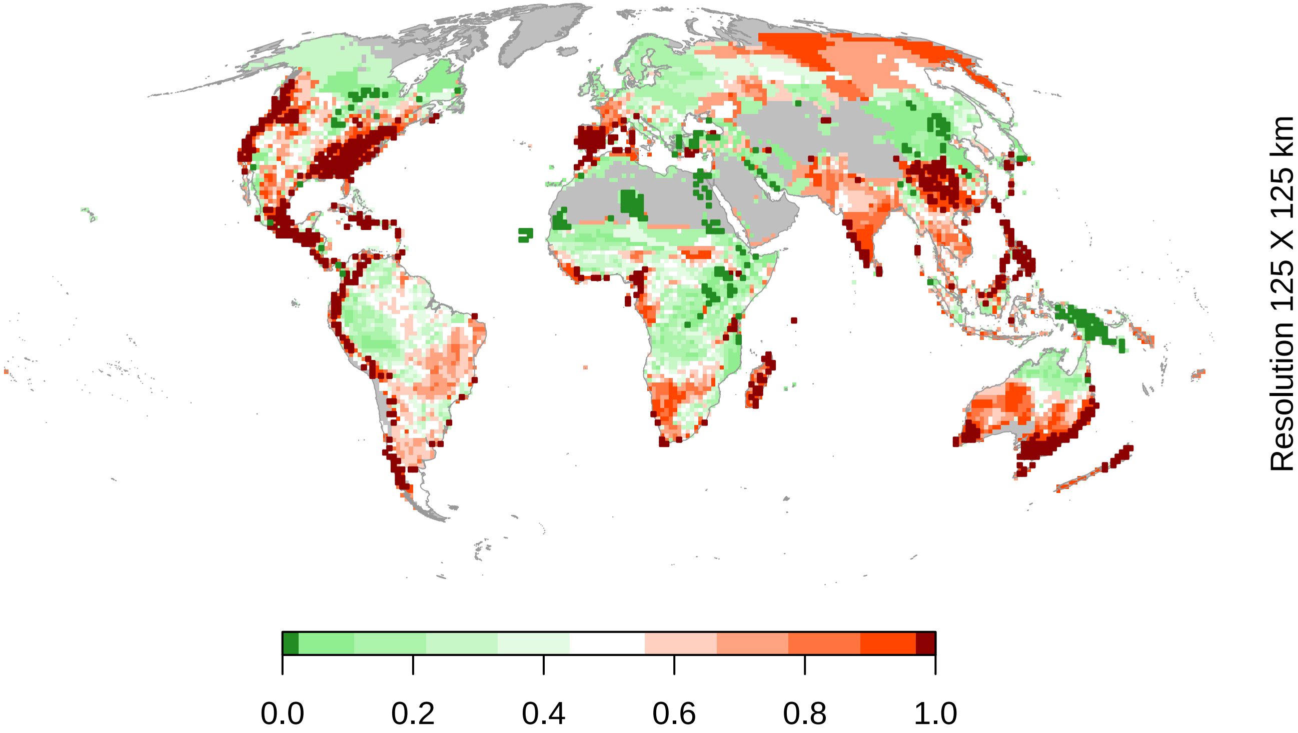

Amphibian ED dispersion

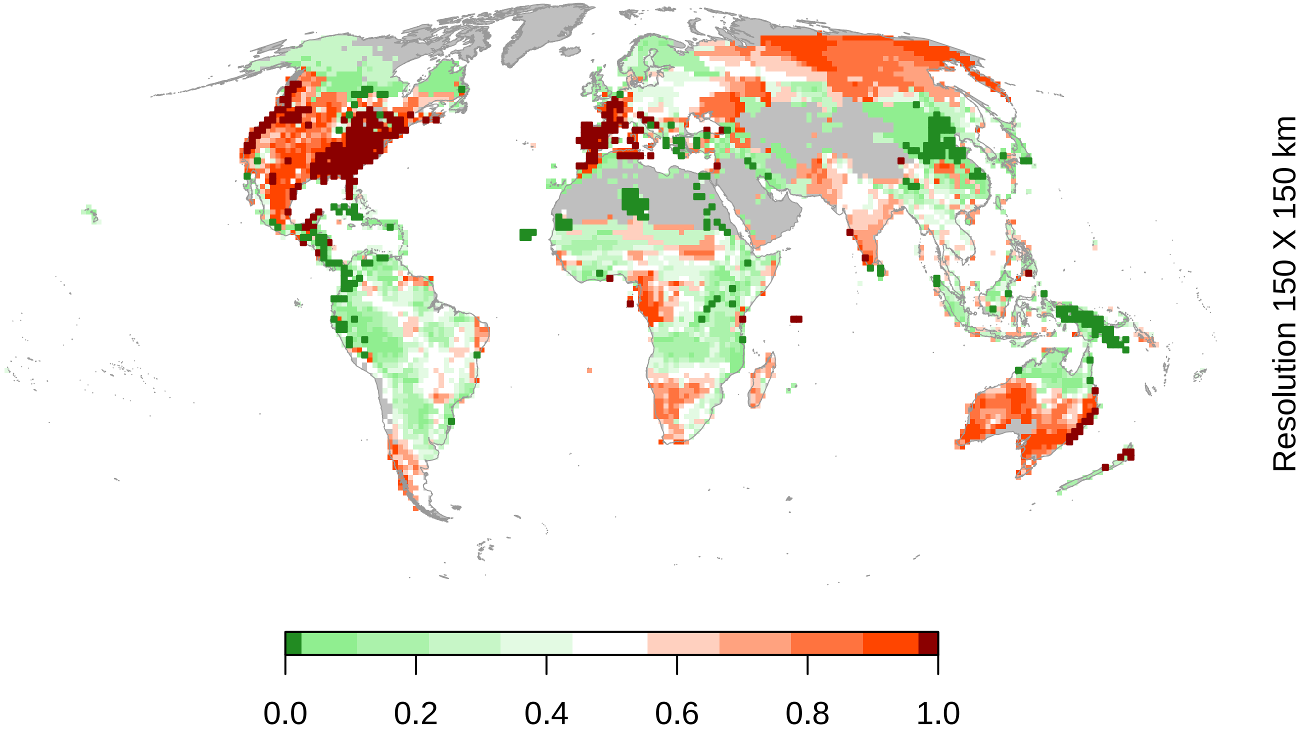

Amphibian EDGE dispersion

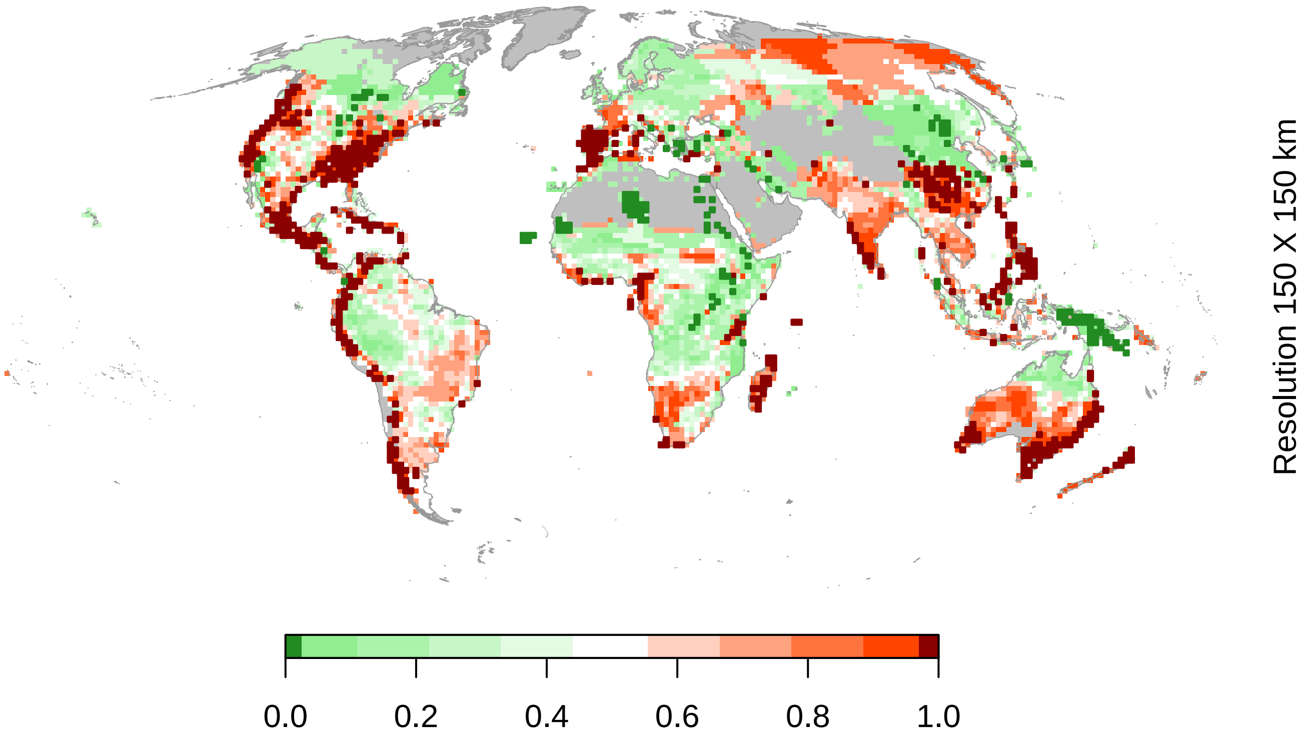

Amphibian ED dispersion

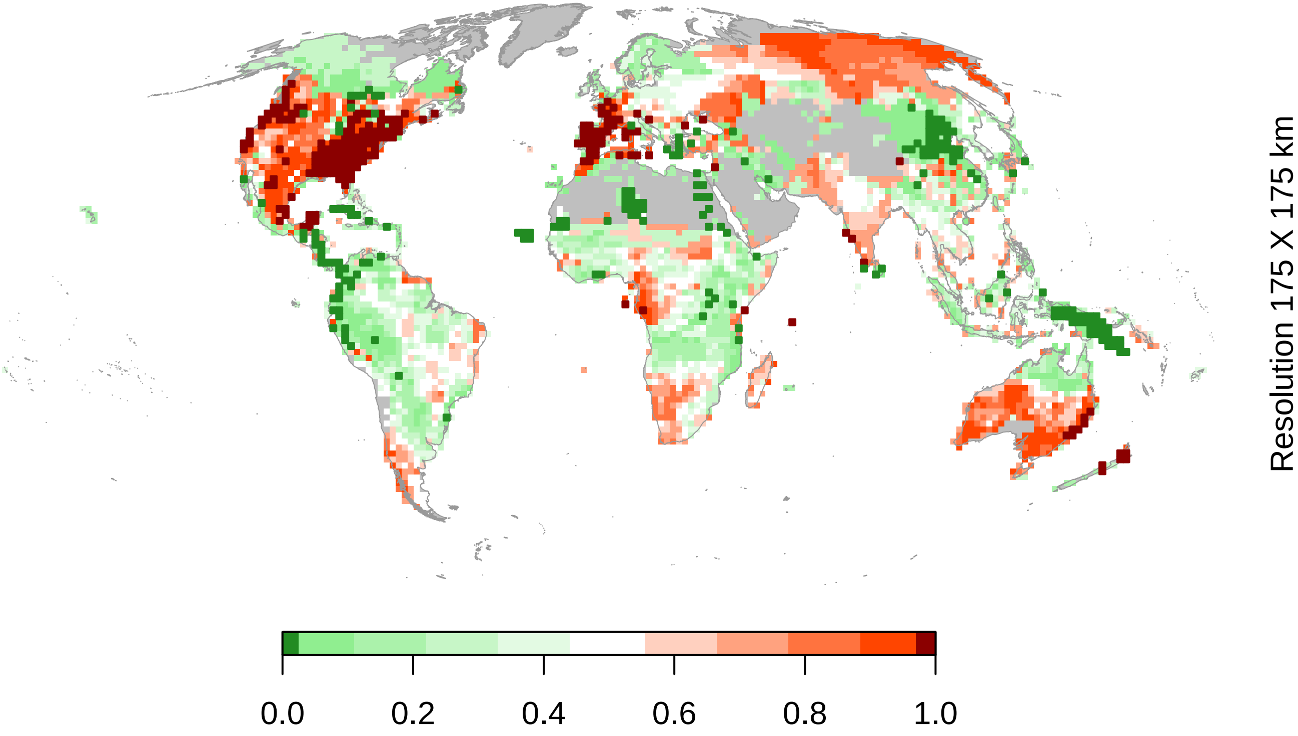

Amphibian EDGE dispersion

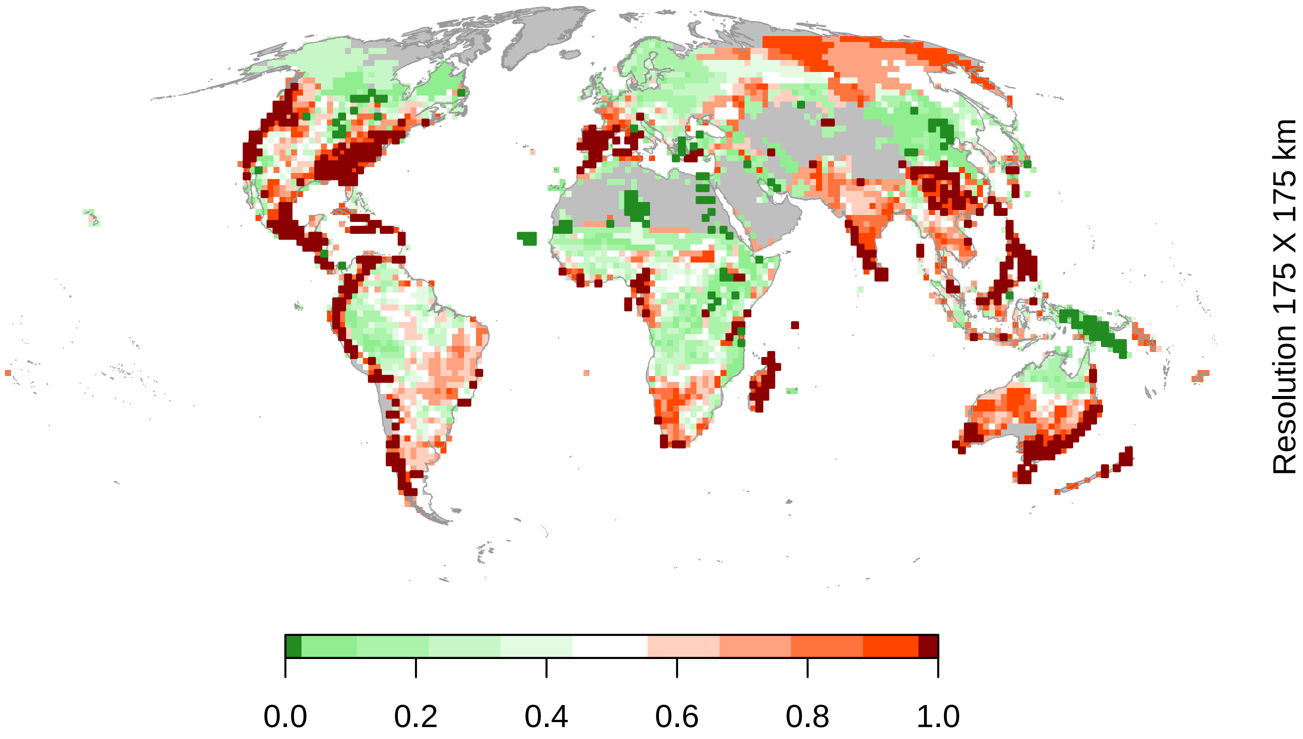

Amphibian ED dispersion

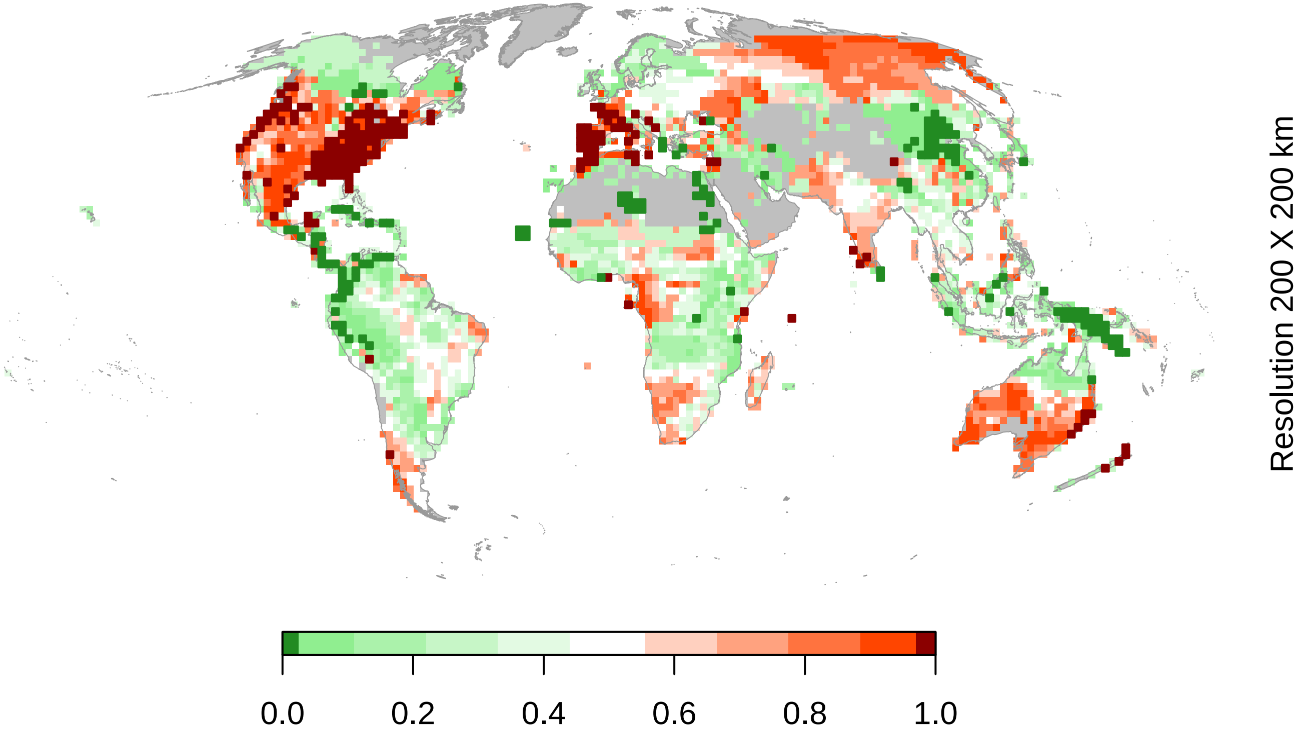

Amphibian EDGE dispersion

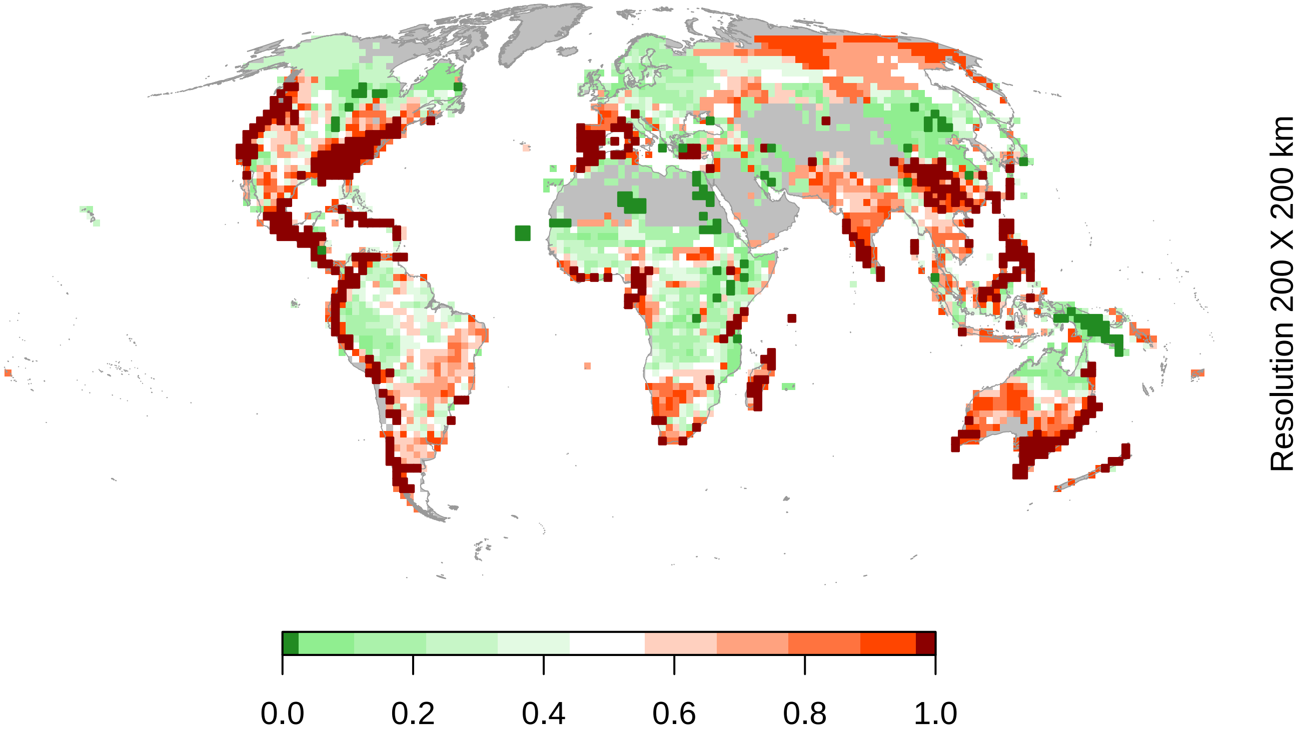

Supplement: Maps S3 — qED and qEDGE maps for amphibians for raster resolutions of 25×25 km to 200×200 km in steps of 25 km. (PDF) [file pone.0063582.s008.pdf]
